# Trends and patterns of varicella zoster virus infection in Kilifi: A population-based serosurvey

**DOI:** 10.1371/journal.pgph.0006013

**Published:** 2026-08-18

**Authors:** Antipa K. Sigilai, Caroline N. Mburu, Rose Selim, Rose Ombati, Donald Akech, Boniface Karia, James Tuju, Gaby P. Smits, van Gageldonk P.G.M., Fiona van der Klis, E. Wangeci Kagucia, J. Anthony G. Scott, Adetifa I.M.O.

**Affiliations:** 1 KEMRI-Wellcome Trust Research Programme, Kilifi, Kenya; 2 Department of Infectious Diseases Epidemiology, London School of Hygiene and Tropical Medicine, London, United Kingdom; 3 Centre for Immunology of Infectious Diseases and Vaccines, National Institute of Public Health, and the Environment (RIVM), Bilthoven, The Netherlands; Washington State University, UNITED STATES OF AMERICA

## Abstract

There is limited epidemiologic data on varicella zoster virus (VZV) infections from low- and middle-income countries including Kenya. We aimed to describe the seroepidemiology of VZV in Kilifi, Kenya, where varicella vaccine is not included in the national infant immunization program, in order to generate evidence to inform vaccine policy. We conducted a retrospective serosurvey utilizing archived plasma and serum samples from cross-sectional population-based serosurveys conducted within the Kilifi Health and Demographic Surveillance System between 2009 and 2021. We assayed immunoglobulin G (IgG) for VZV using a validated Luminex multiplex immunoassay and applied a seropositivity cutoff of ≥0.26 International Units per millilitre (IU/mL), as determined by the assay developer. We calculated Bayesian-adjusted age-specific seroprevalence and tested differences in seroprevalence between groups using Chi square. We used a multivariable logistic regression model to estimate associations with VZV IgG antibody seropositivity. We fitted an age-dependent catalytic model to estimate the force of infection (FOI) in children aged 0.5-4, 5–9 and 10–14 years. A total of 2639 samples from children aged <15 years and 546 samples from persons aged ≥15 years were tested. The overall population-weighted seroprevalence of VZV IgG antibodies among children aged 0–14 years was 38.4% (95% CI 27.5-49.5). Age-specific seroprevalence rose from 13.3% (95% CI 5.8-21.6) in children aged 0–4 years to 60.9% (95% CI 45.0-76.2) in those aged 10–14 years. Survey year and age were associated with VZV IgG antibody seropositivity. Children aged 5–9 years had the highest FOI (0.098; 95% CI 0.077-0.120) per susceptible year while mean age of infection was 24.3 years (95% CrI 17.6–30.1). Approximately 40% of individuals entering adulthood in Kenya remain susceptible to VZV infection, suggesting a substantial and underappreciated risk of severe VZV disease in older population including pregnant women. An infant varicella immunization program might avert disease across both paediatric and adult populations.

## Introduction

Primary infection with varicella zoster virus (VZV) causes varicella (chicken pox) which is often benign and self-limiting in healthy individuals, however, it can cause severe disease in neonates, adolescents, pregnant women, adults, and immunocompromised individuals [1–3]. VZV infection in the first 20 weeks of pregnancy is of particular concern because of the risk of varicella congenital syndrome that is linked to fatal foetal malformations [4]. Rarely, varicella may be characterized by skin and soft tissue bacterial infections, pneumonia, otitis media, central nervous system infections, and even death, particularly in immunocompromised individuals [5–8]. Annually, varicella is estimated to cause 4.2 million hospitalizations worldwide out of which approximately 0.1% result in death. VZV can also remain dormant for years in the sensory nerve ganglia, causing herpes zoster (shingles) when reactivated, which mainly affects the elderly and immunocompromised individuals [9]. The global incidence of shingles in adults aged ≥70 years is estimated to be 918.2 per 100,000 person years [10].

Existing population-based serological studies on VZV are predominantly from high income countries (HICs) where routine childhood varicella vaccination is common [11]. Most of these studies have consistently reported a high seroprevalence of ≥90% by entry to adolescence, primarily driven by childhood vaccination [12–14]. The widespread adoption of universal varicella vaccination in national immunization programs in many HICs has been linked to reduced disease burden and hospitalisation. In Europe for instance, it is estimated that there would be 5.5 million varicella cases every year with up to 3.9 million primary care visits if there were no routine varicella immunization programs [15]. In contrast, VZV infection and the potential benefits of varicella vaccine have received little attention in many low- and middle-income countries (LMICs) despite evidence linking VZV infection to missed school days among infected children and significant loss of productivity among caregivers as they provide care to their sick children [16].

The few studies on the epidemiology of VZV in sub-Sahara Africa (sSA) are limited to hospitalised patients, immunocompromised individuals, refugee camp residents, health workers, and pregnant women [17–20]. In these studies, the seroprevalence of VZV in children below 5 years of age has been reported to be as low as 8% in the Democratic Republic of Congo [21], 24% in hospitalised Kenyan children aged 1–12 years [22], 45% in children aged 1–4 years in Guinea Bissau [23], and 66% in 4–15-year-old children in Nigeria [24]. None of these studies has examined the seroprevalence of VZV and its transmission dynamics among healthy individuals across all age groups at the population level. Understanding population-level VZV immunity and its transmission dynamics can quantify past infections, assess potential risks of outbreaks, and guide country-specific policies to control varicella virus disease. The current study aimed to determine age-specific immunity profiles for VZV in healthy children and adults, underlying transmission patterns, and associated risk factors in Kenya.

## Materials and methods

### Ethics statement

Ethical approval to conduct this study was obtained from the Scientific and Ethics Review Unit (SERU) of the Kenya Medical Research Institute (SERU 3847). The surveys contributing the samples tested as part of this study also received approvals from SERU (SSC 1433, SERU 4085, and SSC 1131). All participants in these serosurveys gave individual written informed consent for residual samples to be stored and be used in future research.

### Archived samples

We obtained archived plasma and serum samples from three serosurveillance studies that were conducted within the Kilifi Health and Demographic Surveillance System (KHDSS) between 2009 and 2021. The KHDSS is located along the Kenyan coastline and has records of births, pregnancies, migration, and deaths that have been collected routinely since 2000 when it was established [25]. The archived samples were collected biennially as part of malaria cross-sectional surveys conducted between 2009 and 2013 (3 total surveys), annually as pneumococcal serosurveys conducted between 2015 and 2019 (3 total surveys), and between December 2020 to May 2021 as part of a COVID-19 serosurvey. Detailed descriptions of the methodologies used in these surveys have been previously published [26–28].

The malaria cross-sectional surveys enrolled samples of about 500 children aged 0–14 years obtained from randomly selected households in mapped clusters for each survey year. Each of the pneumococcal serological surveys enrolled a cross-sectional, age-stratified, simple random sample of 50 children in each of 10 age categories (i.e., 0, 1, 2, 3, 4, 5, 6, 7, 8–9, and 10–14 years) derived from the Kilifi HDSS database. The COVID-19 serosurvey included a cross-sectional, age-stratified, simple random sample of 850 children and adults from the Kilifi HDSS; 100 children were sampled in each five-year age band below 15 years of age, 50 individuals in each five-year age band between 15 and 64 years of age, and 50 adults aged ≥65 years. The archived samples were de-identified and linked to the metadata using unique identifiers. Therefore, all the investigators had no access to information during or after the study that could link individual participants to the samples.

### Harmonization of serological data across surveys

Plasma or serum samples were obtained from three survey frameworks: (i) the Malaria Cross-Sectional Survey (2009–2013), which was conducted annually as an independent, random, cross-sectional, sample among a rolling cohort of healthy children resident in the KHDSS - since 1998. Children recruited into this cohort followed until age 15 [27]. (ii) The Pneumococcal Conjugate Vaccine Impact Study, i.e., PCVIS (2015–2019), was conducted annually using cross-sectional surveys of KHDSS children aged 0–15 years [26]. (iii) The COVID-19 serosurveillance study conducted in 2021 in response to the pandemic randomly sampled residents of the KHDSS across all ages [28]. Although these surveys were conducted for different primary objectives, all were nested within the KHDSS and used population-based sampling frameworks. Participants were selected independently of VZV infection status, and archived serum samples were subsequently tested for VZV IgG antibodies. To improve comparability across survey years, seroprevalence estimates were standardized to a common age structure using multilevel regression and post-stratification.

### Laboratory analyses

Samples were tested for VZV immunoglobulin G (IgG) antibody using a fluorescent-bead-based multiplex immunoassay on the Luminex MAGPIX System, alongside six other antigen targets: diphtheria, pertussis, tetanus, measles, mumps, and rubella. This immunoassay was developed and validated by the National Institute of Public Health, and the Environment (RIVM), in The Netherlands [29,30]. Samples were retrieved between 15/08/2021 and 31/01/2023 and testing was conducted at the KEMRI-Wellcome Trust Research Programme Laboratories in Kilifi.

The VZV antigen, VZ-10 ZV strain (Genway GWB-23D26F, California U.S.A), was coupled to coloured magnetic beads. The samples, standards, and controls were diluted in Phosphate-Buffered Saline containing 0.1% Tween 20 and 3% Bovine Serum Albumin. The samples were then diluted in duplicates at ratios of 1:200 and 1:4000. Each plate included in-house controls, blanks, and standards. We used the international rubella standard serum RUBI-1–94 (NIBSC, Potters Bar, United Kingdom) as a reference, which contains a VZV-specific IgG concentration of 22 international units per millilitre (IU/mL). Antibody concentrations were determined by interpolation into a 5-parameter fit standard curve using Milliplex Analyst version 3.55. Antibody levels were expressed in IU/mL; a seropositivity threshold of ≥0.26 IU/mL as determined by RIVM was applied [31].

### Statistical analyses

We first calculated the overall crude seroprevalence stratified by survey type, age, and sex. To enable comparison of seroprevalence across survey years while accounting for age distribution differences, we standardised our estimates by applying multilevel regression and post-stratification (MLRP) adjusted for specificity of the assay. We used the average population structure from the entire series as our standard population for each of the yearly samples to standardize both the age-specific seroprevalence values as well as the total KHDSS population values (S1 Table). The sensitivity of the assay was given as 100% and specificity was 91.3% [32]. To enable comparison of seroprevalence across survey years while accounting for differences in age distributions, we applied multilevel regression and post-stratification (MRP) adjusted for assay performance [33,34]. A Bayesian hierarchical logistic regression model with a logit link function was fitted with age-specific random effects and a binomial likelihood. Assay sensitivity and specificity were incorporated into the model using validation data from the assay evaluation study, which reported an estimated sensitivity of 100% and specificity of 91.3%, allowing uncertainty in test performance to be propagated through the analysis [33]. Diffuse Normal (0,10000) priors were specified for the intercept and mean age effect, Uniform(0,1) priors for assay sensitivity and specificity, and a half-normal prior (standard deviation = 0.5) for the age-effect standard deviation parameter. Posterior distributions were estimated using Markov chain Monte Carlo methods implemented in rjags (version 4.3.1). Age-specific prevalence estimates were subsequently post-stratified to the average population structure across all survey years to obtain age- and test-adjusted seroprevalence estimates and corresponding 95% credible intervals [33]. Geometric Mean Concentration (GMCs) of VZV IgG antibodies per year and across all ages and their corresponding 95% CI were also calculated and visualised using boxplots.

The samples were grouped by age as follows: 0–4, 5–9, 10–14, 15–24, 25–34, 35–44, 45–54, 55–64, and ≥65 years. Differences in seroprevalence across age groups, sex, and survey year were assessed using the Chi-square test. Independent t-test and one-way analysis of variance (ANOVA) tests were used to determine if there were any significant differences in participants’ mean age by sex, survey years, and type of survey. A multivariate logistic regression model with age, sex, and survey years as independent variables was used to assess risk factors associated with VZV IgG antibody seropositivity.

### Determining age-specific force of infection, basic reproduction number(R_0_) and herd immunity threshold

We used a simple catalytic model to estimate the age-specific force of infection (FOI), λ(a), a measure of the incidence of infection in a susceptible population for three age groups: 0.5-4, 5–9, and 10–14 years between 2009 and 2021. Infants who were 6 months or younger were assumed to be protected by maternal antibodies in line with previous FOI studies for varicella [35]. To avoid this confounding factor, data for children aged ≤6 months were excluded from the FOI analysis. The model shown below follows individuals from birth and assumes a constant FOI over time independent of age.


$$\begin{array}{c} Z(a)=\text{1}-\text{ex}p-\int_{\text{0}}^{a}(\lambda u)du \end{array}$$

We extended the model to allow for age-varying FOI, and the equations below were modelled:


$$\begin{array}{c} Z(a)=\text{1}-\text{exp}\left(-\lambda_{\text{1}}a\right)for\;\text{0}.\text{5}\leq a<\text{5} \end{array}$$

$$\begin{array}{c} Z(a)=\text{1}-\text{exp}\left(-\lambda_{\text{1}}(\text{5}-\text{0}.\text{5})-\lambda_{\text{2}}(a-\text{5})\right)for\;\text{5}\leq a<\text{1}\text{0} \end{array}$$

$$\begin{array}{c} Z(a)=\text{1}-exp\left(-\lambda_{\text{1}}(\text{5}-\text{0}.\text{5})-\lambda_{\text{2}}(\text{1}\text{0}-\text{5})-\lambda_{\text{3}}(a-\text{1}\text{0})\right)for\;\text{1}\text{0}\leq a<\text{1}\text{5} \end{array}$$

Where Z(a) is the proportion seropositive at age a while λ_1_, λ_2_, λ_3_ are the age-dependent FOIs. To estimate transmission intensity across the full age range, we extended the model to adults using the 2021 serosurvey by stratifying data into the following age bands: 15–24, 25–34, 35–44, 45–54, 55–64, and ≥65 years. In this extended formulation, the force of infection was assumed piecewise constant within each age interval, such that;


$$\begin{array}{c} \lambda (a)=\lambda_{j}\;for\;a_{j-\text{1}}\leq a<a_{j} \end{array}$$

and


$$\begin{array}{c} Z(a)=\text{1}-\text{ex}p-\int_{\text{0}}^{a}(\lambda u)du \end{array}$$

We assumed a closed population, lifelong immunity after varicella infection, and that mortality for infected individuals was similar to that of susceptible individuals. The catalytic model was fitted to the age-specific seroprevalence data within a Bayesian framework using a binomial likelihood and independent Uniform(0,1) priors for each age-specific force of infection parameter. Posterior distributions were estimated using Markov chain Monte Carlo (MCMC) methods implemented in the rjags package (version 4.3.1), with Gibbs sampling used as the underlying algorithm for posterior sampling. Four parallel MCMC chains were run following an adaptation period of 1,000 iterations, and posterior samples were obtained from 10,000 iterations per chain. Convergence was assessed using trace plots and the Gelman–Rubin diagnostic ($\hat{R}$ < 1.01), and model fit was evaluated by visual comparison of observed and model-predicted age-specific seroprevalence estimates.

We estimated the basic reproduction number ($R_{\text{0}}$) using the standard endemic relationship between the mean age at infection and transmissibility [36]. Specifically, we combined age-specific forces of infection estimated from the pooled childhood catalytic model (0–14 years, 2009–2021) with those estimated from the adult catalytic model fitted to the 2021 serosurvey (≥15 years) to reconstruct a piecewise constant force of infection profile over the full age range. The implied mean age at infection (A) was computed as;


$$\begin{array}{c} A=\frac{\int_{\text{0}}^{\infty }a\;\lambda (a)S(a)\;da}{\int_{\text{0}}^{\infty }\lambda (a)S(a)\;da},\text{\textasciitilde{}where\textasciitilde{}}S(a)=\text{exp}\left(-\int_{\text{0}}^{a}\lambda (u)\;du\right)\text{.} \end{array}$$

The basic reproduction number was approximated as $R_{\text{0}}=L/A$, where L is the average life expectancy at birth over the study period, 61.85 years. The basic reproduction number ($R_{\text{0}}$) represents the average number of secondary infections generated by a typical infectious individual in a fully susceptible population. Under endemic equilibrium assumptions, $R_{\text{0}}\;$is inversely related to the mean age at infection (A), such that populations with higher transmission intensity experience infection earlier in life, resulting in a lower mean age at infection and a higher $R_{\text{0}}.\;$Conversely, delayed acquisition of infection is associated with lower transmission intensity and smaller $R_{\text{0}}\;$values. Uncertainty in $R_{\text{0}}$was propagated by Monte Carlo sampling from the posterior distributions of the age-specific forces of infection. From this estimate, we were also able to calculate the herd immunity threshold (HIT) for varicella which is the proportion that would need to be immunised to eradicate endemic transmission of the disease under homogeneous transmission. The HIT was calculated as


$$\begin{array}{c} HIT=\text{1}-\text{1}/R_{\text{0}} \end{array}$$

As a sensitivity analysis, we fitted an alternative catalytic model formulation to assess the robustness of the estimated force of infection to modelling assumptions. We extended the current model to allow for waning of immunity following natural infection by including an additional waning parameter with a weakly informative prior (uniform, 0–10% per year) informed by limited published estimates from zoster modelling studies [37]. The model was fitted using the same Bayesian framework and likelihood specification as the primary analysis.

## Results

### Sociodemographic characteristics of the participants

A total of 2639 samples from children aged <15 years and 546 samples from individuals aged ≥15 years were available for analysis. The number of samples obtained from the malaria cross-sectional surveys were 1072 (33.7%), 1283 (40.3%) from the pneumococcal serosurveys, and 830 (26.0%) from the COVID-19 serosurvey. There was no significant difference in the mean age by sex for all survey years except for the 2021 COVID-19 serosurvey. A summary description of distribution of participants’ mean age and sex per each type of serosurvey across all survey years is presented in Table 1.

**Table 1:** Sociodemographic characteristics and distribution of the participants by each survey type, and sex between 2009 and 2021.

Survey year/sociodemographic characteristics	All N (%)	Age mean (SD)	p-value
**Malaria cross-sectional survey**	1072 (33.7) *	**6.2 (3.5)**	
2009	**363 (100)**	**5.1 (2.7)**	
Female	178 (49.0)	5.2 (2.6)	
Male	185 (51.0)	5.0 (2.8)	0.589
2011	**304 (100)**	**6.5 (3.4)**	
Female	149 (49.0)	6.7 (3.4)	
Male	155 (51.0)	6.2 (3.5)	0.219
2013	**405 (100)**	**6.9 (3.9)**	
Female	196 (48.4)	6.9 (3.9)	
Male	209 (51.6)	6.9 (3.8)	0.944
**Pneumococcal biennial serosurvey**	1283 (40.3) *	**5.1 (3.5)**	
2015	**373 (100)**	**5.1 (3.6)**	
Female	170 (45.6)	5.2 (3.6)	
Male	203 (54.4)	5.1 (3.7)	0.672
2017	**417 (100)**	**5.2 (3.4)**	
Female	211 (50.6)	5.4 (3.4)	
Male	206 (49.4)	5.0 (3.4)	0.254
2019	**493 (100)**	**5.0 (3.4)**	
Female	247 (50.1)	5.1 (3.4)	
Male	246 (49.9)	5.0 (3.5)	0.687
**COVID-19 serosurvey**	830 (26.0) *	**30.1 (21.7)**	
2021	830 (100)	30.1 (21.7)	
Female	445 (53.6)	33.5 (21.4)	
Male	385 (46.4)	27.3 (21.7)	<0.001
**Total participants**	**3185 (100)**		


*The values are presented as n (%) or mean (SD). Proportion were compared using Pearson’s Chi-square test and mean age was compared using independent samples t-test

### Age-stratified VZV immunity profiles across survey years

**Fig 1:**
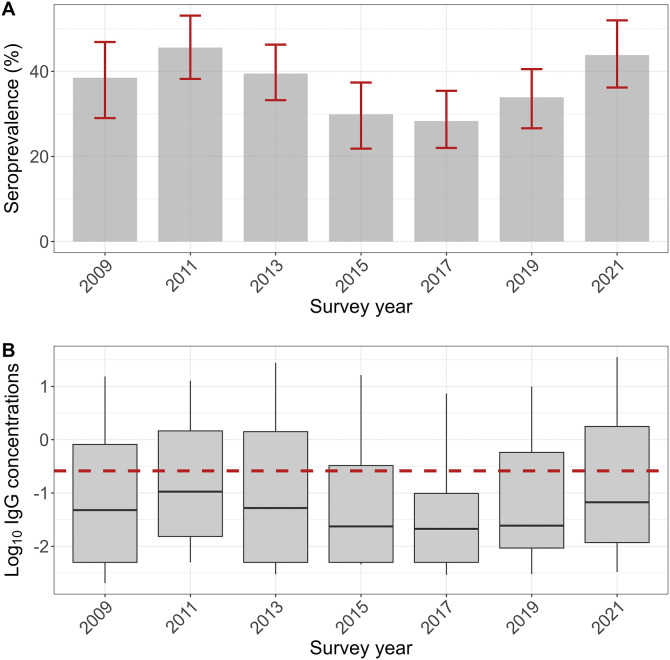
**(A)** **Mean anti-VZV IgG seroprevalence among children aged 0–14 years by survey year. Bars represent seroprevalence estimates and red error bars indicate 95% confidence intervals.** **(B)** Distribution of individual log10-transformed anti-VZV IgG concentrations by survey year. The horizontal line within each box represents the median, the box represents the interquartile range (IQR), and the whiskers indicate the extreme observations. The dashed red line indicates the log10-transformed seropositivity threshold corresponding to 0.26 IU/mL. Geometric mean concentrations (GMCs) reported in the text are presented on the original IU/mL scale, whereas Panel B displays individual anti-VZV IgG concentrations on the log10-transformed scale for visualization.

Bayesian age-standardised seroprevalence of VZV IgG antibodies for children aged 0–14 ranged from 28.3% (95% CI 22.0-35.4) in 2017 to 45.6% (95% CI 38.2-53.1) in 2011. This pattern demonstrates existing variation in proportion of children considered immune to VZV across survey years as shown in Fig 1A. A summary of the Bayesian population-weighted and test-adjusted anti-VZV IgG seroprevalence for each survey year is provided in S2 Table. The geometric mean concentrations (GMCs) of VZV IgG antibodies, calculated on the original IU/mL scale, showed a cyclic pattern with an initial increase from 0.07 (95% CI 0.06–0.10) IU/mL in 2009 to 0.13 (95% CI 0.10–0.18) IU/mL in 2011. This was followed by a decline to the lowest concentration of 0.04 (95% CI 0.03–0.05) IU/mL in 2017. However, there was a subsequent increase to 0.05 (95% CI 0.04–0.07) IU/mL in 2019 and 0.14 (95% CI 0.11–0.20) IU/mL in 2021. Consistent with these GMC estimates, Fig 1B shows the distribution of log-transformed VZV IgG concentrations across survey years, with higher median antibody levels observed in 2011 and 2021 than in 2015–2019 (Fig 1B).

**Fig 2:**
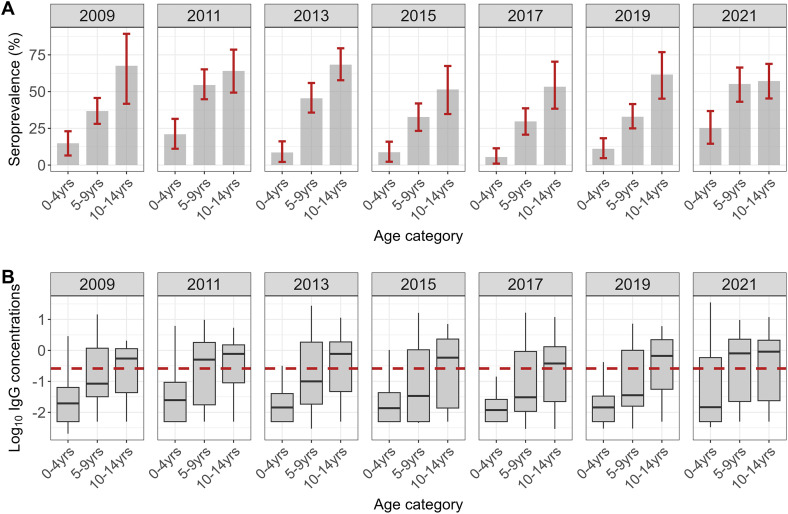
**(A)** **Anti-VZV IgG seroprevalence among children aged 0–14 years by age category and survey year. Bars represent seroprevalence estimates and red error bars indicate 95% confidence intervals.** **(B)** Distribution of individual log10-transformed anti-VZV IgG concentrations by age category and survey year. The horizontal line within each box represents the median, the box represents the interquartile range (IQR), and the whiskers indicate the extreme observations. The dashed red line indicates the log10-transformed seropositivity threshold corresponding to an anti-VZV IgG concentration of 0.26 IU/mL.

Across all the survey years, the Bayesian age-standardised seroprevalence of VZV IgG antibodies was consistently lowest among children aged 0–4 years, ranging from 5.5% (95% CI 1.0-11.4) in 2017 to 25.3% (95% CI 14.5-36.8) in 2021. In contrast, seroprevalence among children aged 5–9 years ranged from 29.7% (95% CI 20.7-38.6) in 2017 to 55.2% (95% CI 43.1-66.4) in 2021. The highest seroprevalence levels were consistently observed among children aged 10–14-year-olds across survey years with a low of 51.4% (95% CI 34.7-67.4) in 2015 and a peak of 68.3% (95% CI 57.7-79.4; Fig 2A) in 2013.

Similarly, the mean GMCs VZV IgG concentration across all survey years showed an increase with age from 0.03 (95% CI 0.02-0.03) IU/mL among children <5 years to 0.12 (95% CI 0.10-0.13)IU/mL in 5–9-year-olds and reaching 0.28 (95% CI 0.22-0.34) IU/mL in those aged 10–14 years]. Overall, the median IgG concentrations of all children <5 years were below seropositivity threshold while those in the 10–14-year group remained consistently above the seropositivity threshold across all survey years. Children aged 5–9 years showed variable findings with the median VZV IgG concentration reaching above seropositivity threshold only in 2011 and 2021 (Fig 2B). A similar pattern was observed in older children and adults aged ≥15 years in the 2021 serosurvey (S1A and S1B Fig).

### Determinants of VZV seropositivity in children

In our multivariate logistic regression model, age was significantly associated with VZV IgG antibody seroprevalence. Compared to children aged 0–4 years, all other age groups had significantly higher odds of testing positive for VZV IgG antibodies (adjusted odds ratios [aORs] 3.71 – 21.92; p < 0.001). Using 2009 as our baseline survey year, the odds of seropositivity were significantly higher in 2011 (aOR= 1.57; 95% CI 1.12-2.19) and 2021 (aOR= 1.48; 95% CI: 1.04-2.09) and lower in 2017 (aOR= 0.65; 95% CI 0.47-0.91; Table 2).

**Table 2:** A multivariate logistic model for risk factors of VZV seropositivity in children aged 0-14 years.

Independent variables	Partici-pants N	Seropositive n (%)	Unadjusted Odds Ratio (95% CI)	*p-value*	Adjusted Odds Ratio (95% CI)	*p-value*
Age (years)						
0-4	1059	161 (15.1)	1		1	
5-9	1130	456 (40.2)	3.79 (3.09-4.65)	<0.001	3.71 (3.02-4.57)	<0.001
10-14	450	272 (60.6)	8.66 (6.73-11.15)	<0.001	7.64 (5.90-9.90)	<0.001
15-24	105	72 (68.6)	12.28 (7.87-19.16)	<0.001	8.06 (4.88-13.33)	<0.001
25-34	100	71 (71.0)	13.78 (8.67-21.90)	<0.001	9.00 (5.32-15.11)	<0.001
35-44	98	75 (76.5)	18.35 (11.17-30.14)	<0.001	11.87 (6.83-20.62)	<0.001
45-54	97	82 (84.5)	30.76 (17.30-54.70)	<0.001	19.96 (10.70-37.24)	<0.001
55-64	98	84 (85.7)	33.76 (18.71-60.91)	<0.001	21.92 (11.59-41.47)	<0.001
>=65	50	42 (84.0)	29.54 (13.62-64.09)	<0.001	19.26 (8.56-43.32)	<0.001
Survey year						
2009	363	114 (31.4)	1		1	
2011	304	141 (46.4)	1.89 (1.38-2.59)	<0.001	1.57 (1.12-2.19)	0.009
2013	407	169 (41.5)	1.55 (1.15-2.08)	0.004	1.17 (0.85-1.61)	0.338
2015	373	96 (25.7)	0.76 (0.55-1.04)	0.089	0.76 (0.54-1.07)	0.114
2017	417	100 (24.0)	0.69 (0.50-0.94)	0.021	0.65 (0.47-0.91)	0.012
2019	493	138 (28.0)	0.85 (0.63-1.13)	0.262	0.86 (0.63-1.17)	0.342
2021	284	132 (46.5)	1.90 (1.38-2.61)	<0.001	1.48 (1.04-2.09)	0.028

### Force of Infection (FOI)

**Fig 3:**
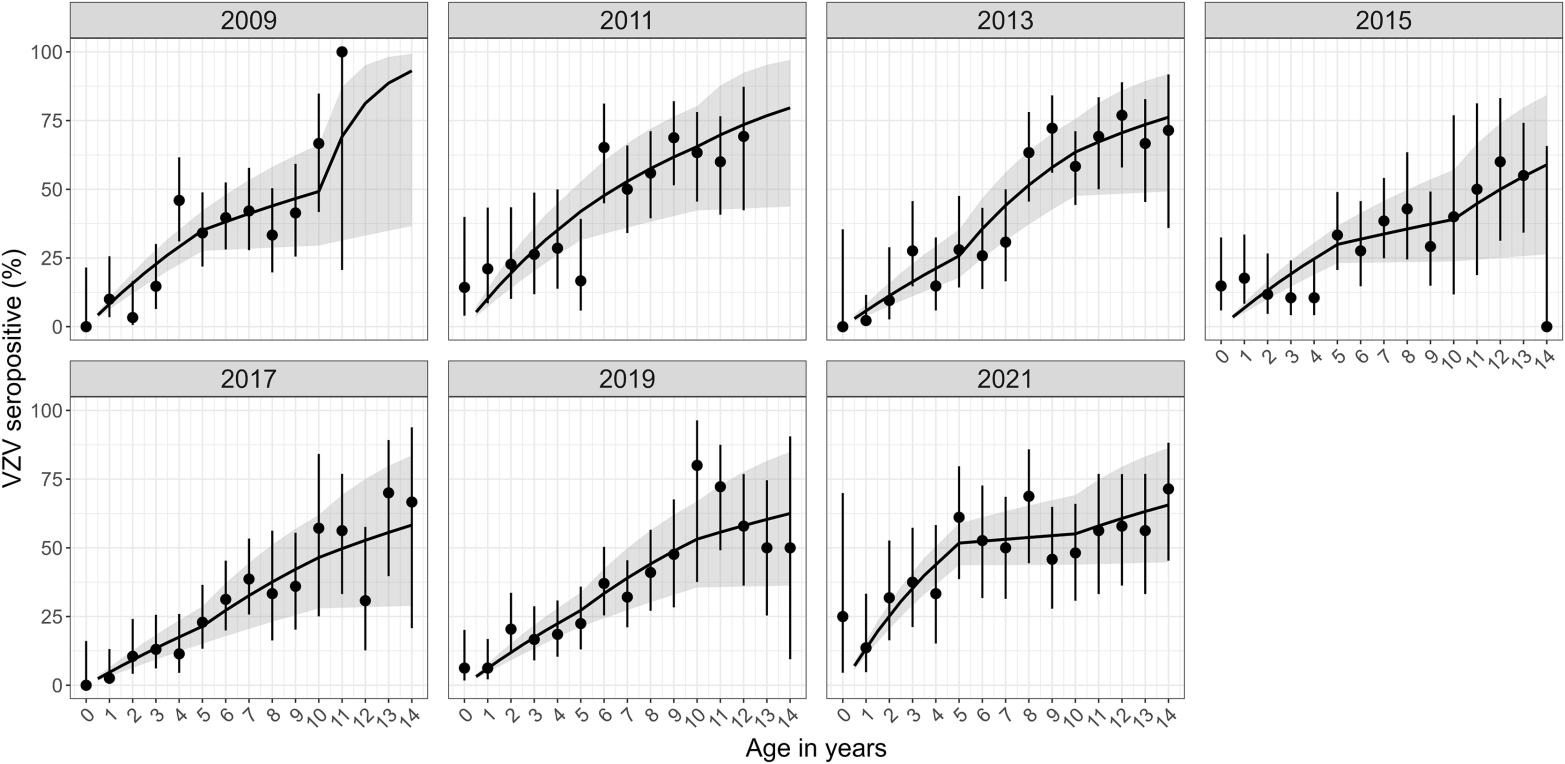
Observed and model-predicted anti-VZV IgG seroprevalence among children aged 0–14 years in Kilifi by survey year (2009–2021) without waning. Black points represent the observed seroprevalence estimates at each age, and black vertical lines indicate their corresponding 95% binomial confidence intervals. The solid black line represents the median model-predicted seroprevalence, and the grey shaded region represents the 95% credible interval of the model predictions.

Both the age-dependent catalytic models in children and adults successfully converged and captured the seroprevalence trends in both children (Fig 3) and adults (S4 Fig). Among children aged <15 years, the estimated annual rate of infection per susceptible individual was highest among those aged 5–9 years at 0.09 (95% CI 0.07-0.11) followed by children aged 0.5-4 years at a rate of 0.07 (95% CI 0.06-0.08) and lowest in children aged 10–14 years at a rate of 0.04 (95% CI 0.01-0.11). This corresponds to a decrease in the reproduction number from 5.75 (95% CI 4.15-6.83) in 5–9-year-olds, to 4.47 (95% CI 4.21-5.36) in under 5-year-olds and further to 3.07 (95% CI 0.25-6.83) in children between 10 and 14 years of age. The median FOI in participants who were 15 years or older was lower than 0.05 in all the age groups indicating relatively weak transmission in adulthood (Table 3). The implied mean age at infection from the combined pooled childhood (0–14 years) and adult (≥15 years, 2021) force of infection estimates was 24.3 years (95% CrI 17.6–30.1), reflecting delayed acquisition of varicella and substantial residual susceptibility into adulthood. This corresponded to an estimated basic reproduction number of $R_{\text{0}}=\text{2}.\text{5}\text{4}$ (95% CrI 2.06–3.52). The associated herd immunity threshold was 61% (95% CrI 52%–72%).

**Table 3:** Force of infection by age estimated from the catalytic model.

Age Group (Years)	Median	95% CI
0-4	0.07	[0.06-0.08]
5-9	0.09	[0.07-0.11]
10-14	0.04	[0.01-0.11]
15-24	0.02	[0.00-0.05]
25-34	0.01	[0.00-0.05]
35-44	0.02	[0.00-0.07]
45-54	0.04	[0.00-0.07]
55-64	0.04	[0.00-0.15]
≥65	0.03	[0.00-0.11]

**Note**: Force of infection estimates for children aged 0–14 years were obtained from a catalytic model fitted to pooled serological data from all survey years (2009–2021). Force of infection estimates for participants aged ≥15 years were obtained from a separate catalytic model fitted to the 2021 population-wide serosurvey.

Inclusion of waning of VZV immunity had minimal impact on the estimated forces of infection across age groups, with FOI estimates remaining highly similar to those obtained under the primary model without waning (S2 and S3 Figs). The posterior median waning rate was 0.087 per year (95% CrI 0.037–0.100). Within the reversible catalytic model, this parameter represents the annual rate of transition from the seropositive to the seronegative state and is therefore interpreted as the rate of loss of seropositivity. However, the posterior distribution of the waning parameter was broad, indicating that waning was weakly identifiable from these data.

## Discussion

We used representative samples of serological specimens from a biobank to characterize the epidemiology of VZV infection in Kilifi over a span of more than 10 years. Our findings showed that about 38% of children under the age of 15 years had serological evidence of prior VZV infection. VZV IgG antibody seroprevalence increased significantly from about 15% in children aged 0–4 years to 60% among adolescents aged 10–14 years and to >80% among adults aged ≥45years. In contrast, studies in high-income, temperate settings have reported much higher infection-induced VZV seroprevalence. For instance, in the Netherlands infection-induced VZV seroprevalence is about 95% by the age of 6 years [38]. This mirrors findings prior to the introduction of an infant varicella immunization program in Germany where VZV seroprevalence was about 86% among children aged 6 years and 94% among those aged 17 years [39].

Still, our findings were comparable with reports from other tropical and sub-tropical countries in the absence of a routine varicella vaccination. For instance, the seroprevalence of VZV IgG antibodies was 50% among Thai children aged 10–14 years and 60% in Singaporean children aged 7–12 years. In Sri Lanka, only 20% of children aged 1–10 years had evidence of VZV infection and 25% of adults aged ≥40 years remained at risk of VZV infection [40]. A similar pattern was observed in other tropical and subtropical countries such as Pakistan [41], India [42], and Sudan [43]. We also found that the rate of infection was highest in children aged 5–9 years suggesting higher transmission among primary school-going children. This higher transmission observed in children aged 5–9 years in our model is consistent to what has been reported in countries such as Luxembourg [44], Canada [45], Germany, England and Wales among other high-income countries during the pre-vaccine era [46].

Although we observed age-dependent increases in seropositivity due to repeated exposure to naturally circulating VZV, we found that about 40% of persons aged ≥15 years remained susceptible to VZV infection. This pattern is consistent with delayed acquisition of varicella infection and a substantial shift of infections into adolescence and adulthood, as reflected by the relatively high mean age at infection and moderate R₀ estimated in our study compared with many temperate settings where higher transmission intensity results in infection occurring predominantly during early childhood. Although forces of infection in adults were low, a large proportion of individuals escaped infection in childhood and remained susceptible beyond adolescence, resulting in a prolonged tail of infections occurring gradually at older ages.

Available evidence shows that delayed exposure to VZV infections in late childhood, adolescence, or early adulthood is associated with more severe disease [47]. The sSA region has recorded small to moderate burden of severe VZV illness in adults. Notably, a study from the Democratic Republic of Congo found that of the 124 adults with PCR-confirmed VZV infection, 13–99% had systemic symptoms, 79% had 50 or more lesions, and 15% were bedridden [48]. In Zambia, VZV was found to cause about 4% of central nervous system (CNS) disorders among HIV-positive adults, with a 30% case fatality rate among patients with VZV-induced CNS disorders [49]. The findings from our study could be incorporated into dynamic VZV transmission models which help build the evidence base for the burden of VZV disease in Kenya.

A significant proportion of adolescents and adults in this study remained susceptible to VZV raising concern of increased risk of severe disease and shingles in older age groups. Although evidence from varicella vaccine effectiveness studies suggest routine childhood varicella immunization programs significantly reduce VZV-associated hospitalisation, complications, and mortality [50–55], local disease burden is needed to justify vaccine introduction in Kenya. An infant immunization program for varicella in Kenya may not only confer direct benefits by averting disease in children but may also contribute towards closing the immunity gap among adults. Although we estimated a herd immunity threshold of 61%, this represents a theoretical transmission threshold rather than a vaccination coverage target, as the coverage required to interrupt transmission would additionally depend on vaccine effectiveness, duration of protection, and heterogeneity in contact patterns. As a sensitivity analysis, we explored the potential impact of waning immunity following natural varicella infection. Allowing for waning had minimal effect on the estimated age-specific forces of infection and did not materially alter the overall transmission patterns inferred from the primary analysis. The waning parameter was weakly identifiable from the available cross-sectional serological data, with broad posterior uncertainty, reflecting both limited information in the data and the absence of robust empirical estimates of waning following natural VZV infection in the literature. Although waning could not be reliably quantified in this setting and did not substantially influence inference, waning of serological markers following natural infection remains biologically plausible and is thought to occur over long timescales, as evidenced by age-related declines in VZV-specific cellular immunity and reactivation as herpes zoster [37,56].

### Strengths and limitations

One of the strengths of this study lies in the random sample of the participants that were drawn from a population with known demographic characteristics. Because of this, we were able to adjust the seroprevalence estimates for the underlying population distribution through population-weighting. This population-based random sampling approach improves the internal validity of the findings. Another key strength of this study is the large sample size that allowed for robust trend analyses of VZV seroprevalence across different ages spanning over 10 years. Therefore, this study provides baseline data on susceptibility patterns which could be used to inform the need for and design of a varicella immunization program in Kenya.

Our study design also had few limitations; first samples for this study were obtained in a rural setting in coastal Kenya limiting the generalization of the findings across Kenya. Because VZV transmission is strongly influenced by population density, contact patterns, household structure, and school mixing, force of infection and transmission intensity may differ in urban settings or other regions of the country. For example, higher contact rates in densely populated urban areas could result in higher force of infection, younger ages at infection, and higher estimates of transmissibility than those observed in Kilifi. Second, there were few participants aged <6 months and this limited our understanding of the potential benefits of protection from maternal antibody. An additional limitation is that samples were pooled from non-uniform serosurveys originally conducted for different research purposes. Although all surveys were population-based and nested within the KHDSS, differences in sampling procedures across studies could have introduced residual selection bias. The direction of any resulting bias is difficult to predict because VZV exposure was not a factor in participant selection and could plausibly lead to either overestimation or underestimation of seroprevalence if sampled individuals differed systematically from the underlying population in their cumulative exposure to VZV. Lastly, the combination of imperfect assay and potential unmeasured socioeconomic factors may have introduced bias into our results.

## Conclusion

This study describes the seroepidemiology of VZV in children aged 0–14 years and, to a lesser extent, among older children and adults aged ≥15 years in Kenya. Although evidence from this study shows an increase in anti-VZV IgG seropositivity with age, the FOI is low resulting in about 40% of children transitioning into adulthood without VZV immunity. The observed transmission pattern across all ages reflected continued infection into adulthood, suggesting a hidden burden of severe VZV disease among adolescents and adults including sequelae such as congenital varicella syndrome. Taken together, seroepidemiological and modelling data identify a substantial susceptible gap extending into adolescents and adulthood, suggesting the potential for continued VZV infection at older ages. These findings support the need for additional studies to quantify VZV morbidity burden, potential vaccine impact, its cost-effectiveness, and programmatic feasibility to inform varicella vaccine policy in Kenya.

## Supporting information

S1 Table Mid-year population estimates in KHDSS. The average population across the years was used as the standard population.
(DOCX)

S2 Table Comparison of seroprevalence of anti-VZV IgG antibody among children aged 0–14 years across all years. (DOCX)

S1 Fig (A) Anti-VZV IgG seroprevalence among older children and adults in the 2021 population-wide survey by age group. Bars represent seroprevalence estimates and red error bars indicate 95% confidence intervals. **(B)** Distribution of individual log10-transformed anti-VZV IgG concentrations by age group. The horizontal line within each box represents the median, the box represents the interquartile range (IQR), and the whiskers indicate the extreme observations. The dashed red line indicates the log10-transformed seropositivity threshold corresponding to an anti-VZV IgG concentration of 0.26 IU/mL.
(TIF)

S2 Fig Observed and model-predicted anti-VZV IgG seroprevalence among children aged 0–14 years in Kilifi by survey year (2009–2021) with waning. Black points represent the observed seroprevalence estimates at each age, and black vertical lines indicate their corresponding 95% binomial confidence intervals. The solid black line represents the median model-predicted seroprevalence, and the grey shaded region represents the 95% credible interval of the model predictions.
(TIF)

S3 Fig Comparison of observed and model-predicted anti-VZV IgG seroprevalence among adults by age group in the 2021 population-wide survey without waning for all ages. Black circles represent the observed seroprevalence estimates and black vertical lines indicate the corresponding 95% confidence intervals. Grey squares represent the median model-predicted seroprevalence, and grey vertical lines indicate the corresponding 95% credible intervals.
(TIF)

S4 Fig Comparison of observed and model-predicted anti-VZV IgG seroprevalence among adults by age group in the 2021 population-wide survey with waning for all ages. Black circles represent the observed seroprevalence estimates and black vertical lines indicate the corresponding 95% confidence intervals. Grey squares represent the median model-predicted seroprevalence, and grey vertical lines indicate the corresponding 95% credible intervals.
(TIF)

S1 Checklist STROBE checklist (Strengthening the Reporting of Observational Studies in Epidemiology). Reproduced under the terms of the Creative Commons Attribution 4.0 International (CC BY 4.0) License.
(DOCX)

## References

[1] Gershon AA, Gershon MD. Pathogenesis and current approaches to control of varicella-zoster virus infections. Clin Microbiol Rev. 2013;26(4):728–43. doi: 10.1128/CMR.00052-13 24092852 PMC3811230

[2] Kennedy PGE, Gershon AA. Clinical Features of Varicella-Zoster Virus Infection. Viruses. 2018;10(11):609. doi: 10.3390/v10110609 30400213 PMC6266119

[3] Nagel MA, Bubak AN. Varicella Zoster Virus Vasculopathy. J Infect Dis. 2018;218(suppl_2):S107–12. doi: 10.1093/infdis/jiy425 30247600 PMC6151079

[4] Adams Waldorf KM, McAdams RM. Influence of infection during pregnancy on fetal development. Reproduction. 2013;146(5):R151-62. doi: 10.1530/REP-13-0232 23884862 PMC4060827

[5] Bernal JL, Hobbelen P, Amirthalingam G. Burden of varicella complications in secondary care, England, 2004 to 2017. Euro Surveill. 2019;24(42):1900233. doi: 10.2807/1560-7917.ES.2019.24.42.1900233 31640840 PMC6807256

[6] Marin M, Watson TL, Chaves SS, Civen R, Watson BM, Zhang JX. Varicella among adults: data from an active surveillance project, 1995-2005. J Infect Dis. 2008;197(Suppl 2):S94–100. doi: 10.1086/522155 18419417

[7] van Lier A, van der Maas NAT, Rodenburg GD, Sanders EAM, de Melker HE. Hospitalization due to varicella in the Netherlands. BMC Infect Dis. 2011;11:85. doi: 10.1186/1471-2334-11-85 21466668 PMC3080814

[8] Zou J, Krentz HB, Lang R, Beckthold B, Fonseca K, Gill MJ. Seropositivity, risks, and morbidity from varicella-zoster virus infections in an adult PWH cohort from 2000-2020. Open Forum Infectious Diseases. 2022;9(8):ofac395. doi: 10.1093/ofid/ofac395 PMC9394766 36004318

[9] WHO. Varicella and herpes zoster vaccines: WHO position paper, June 2014. 2014. 24983077

[10] Yang C, Guo Z, Deng X, Wei Y, Zhang Q, Wang J. Global, regional, and national burden of varicella-zoster infections in adults aged 70 years and older from 1997 to 2021: Findings from the Global Burden of Disease Study. J Infect Public Health. 2025;18(10):102868. doi: 10.1016/j.jiph.2025.102868 40616910

[11] Mészner Z, Wysocki J, Richter D, Zavadska D, Ivaskeviciene I, Usonis V, et al. Burden of varicella in Central and Eastern Europe: findings from a systematic literature review. Expert Rev Vaccines. 2019;18(3):281–93. doi: 10.1080/14760584.2019.1573145 30810402

[12] Reynolds MA, Kruszon-Moran D, Jumaan A, Schmid DS, McQuillan GM. Varicella seroprevalence in the U.S.: data from the National Health and Nutrition Examination Survey, 1999-2004. Public Health Rep. 2010;125(6):860–9. doi: 10.1177/003335491012500613 21121231 PMC2966667

[13] Wiese-Posselt M, Siedler A, Mankertz A, Sauerbrei A, Hengel H, Wichmann O, et al. Varicella-zoster virus seroprevalence in children and adolescents in the pre-varicella vaccine era, Germany. BMC Infect Dis. 2017;17(1):356. doi: 10.1186/s12879-017-2461-2 28525973 PMC5438501

[14] Zanella B, Bechini A, Bonito B, Del Riccio M, Ninci A, Tiscione E, et al. A Study of Varicella Seroprevalence in a Pediatric and Adolescent Population in Florence (Italy). Natural Infection and Vaccination-Acquired Immunization. Vaccines (Basel). 2021;9(2):152. doi: 10.3390/vaccines9020152 33672915 PMC7918443

[15] Riera-Montes M, Bollaerts K, Heininger U, Hens N, Gabutti G, Gil A, et al. Estimation of the burden of varicella in Europe before the introduction of universal childhood immunization. BMC Infect Dis. 2017;17(1):353. doi: 10.1186/s12879-017-2445-2 28521810 PMC5437534

[16] Wittenberg R, Damant J, Rehill A, Knapp M, Adeyemi T, Matthews I. Estimated value of productivity lost due to childhood chickenpox in the United Kingdom: a survey of parents. Expert Rev Pharmacoecon Outcomes Res. 2025;25(2):197–203. doi: 10.1080/14737167.2024.2410257 39351785

[17] Hussey H, Abdullahi L, Collins J, Muloiwa R, Hussey G, Kagina B. Varicella zoster virus-associated morbidity and mortality in Africa - a systematic review. BMC Infect Dis. 2017;17(1):717. doi: 10.1186/s12879-017-2815-9 29137604 PMC5686819

[18] Ibrahim EG, Abdel Wahed WY, Eid HM, Deeb WS. Seroprevalence of varicella-zoster virus among pregnant women in Fayoum Governorate, Egypt. J Egypt Public Health Assoc. 2019;94(1):2. doi: 10.1186/s42506-018-0002-5 30686830 PMC6325979

[19] Leung J, Lopez A, Mitchell T, Weinberg M, Lee D, Thieme M, et al. Seroprevalence of varicella-zoster virus in five US-bound refugee populations. J Immigr Minor Health. 2015;17(1):310–3. doi: 10.1007/s10903-013-9946-x 24271111 PMC4606866

[20] Rubaihayo J, Tumwesigye NM, Konde-Lule J. Trends in prevalence of selected opportunistic infections associated with HIV/AIDS in Uganda. BMC Infect Dis. 2015;15:187. doi: 10.1186/s12879-015-0927-7 25879621 PMC4408591

[21] Doshi RH, Alfonso VH, Mukadi P, Hoff NA, Gerber S, Bwaka A, et al. Low Varicella Zoster Virus Seroprevalence Among Young Children in the Democratic Republic of the Congo. Pediatr Infect Dis J. 2018;37(2):138–43. doi: 10.1097/INF.0000000000001750 28834954 PMC5762406

[22] Admani B, Macharia WM, Were F. Seroprevalence of varicella zoster antibodies among children with malnutrition, malignancies and HIV infection. East Afr Med J. 2008;85(10):480–6. doi: 10.4314/eamj.v85i10.9669 19537424

[23] Poulsen A, Cabral F, Nielsen J, Roth A, Lisse IM, Vestergaard BF, et al. Varicella zoster in Guinea-Bissau: intensity of exposure and severity of infection. Pediatr Infect Dis J. 2005;24(2):102–7. doi: 10.1097/01.inf.0000151034.15747.4a 15702036

[24] Bugaje M, Yusuf H, Abdulkadir I, Ahmed A. Seroprevalence of Varicella Zoster Virus Infection among Primary school Children In Northern Nigeria. Nig J Paed. 2011;38(4). doi: 10.4314/njp.v38i4.72278

[25] Scott JAG, Bauni E, Moisi JC, Ojal J, Gatakaa H, Nyundo C, et al. Profile: The Kilifi Health and Demographic Surveillance System (KHDSS). Int J Epidemiol. 2012;41(3):650–7. doi: 10.1093/ije/dys062 22544844 PMC3396317

[26] Gallagher KE, Adetifa IMO, Mburu C, Bottomley C, Akech D, Karani A, et al. Population immunity to pneumococcal serotypes in Kilifi, Kenya, before and 6 years after the introduction of PCV10 with a catch-up campaign: an observational study of cross-sectional serosurveys. Lancet Infect Dis. 2023;23(11):1291–301. doi: 10.1016/S1473-3099(23)00206-2 37429307 PMC7616650

[27] Mwangi TW, Ross A, Snow RW, Marsh K. Case definitions of clinical malaria under different transmission conditions in Kilifi District, Kenya. J Infect Dis. 2005;191(11):1932–9. doi: 10.1086/430006 15871128 PMC3545188

[28] Etyang AO, Adetifa I, Omore R, Misore T, Ziraba AK, Ng’oda MA, et al. SARS-CoV-2 seroprevalence in three Kenyan health and demographic surveillance sites, December 2020-May 2021. PLOS Global Public Health. 2022;2(8). doi: 10.1371/journal.pgph.0000883 PMC10021917 36962821

[29] Smits GP, van Gageldonk PG, Schouls LM, van der Klis FRM, Berbers GAM. Development of a bead-based multiplex immunoassay for simultaneous quantitative detection of IgG serum antibodies against measles, mumps, rubella, and varicella-zoster virus. Clin Vaccine Immunol. 2012;19(3):396–400. doi: 10.1128/CVI.05537-11 22237896 PMC3294611

[30] van Gageldonk PGM, van Schaijk FG, van der Klis FR, Berbers GAM. Development and validation of a multiplex immunoassay for the simultaneous determination of serum antibodies to Bordetella pertussis, diphtheria and tetanus. J Immunol Methods. 2008;335(1–2):79–89. doi: 10.1016/j.jim.2008.02.018 18407287

[31] van Lier A, Smits G, Mollema L, Waaijenborg S, Berbers G, van der Klis F, et al. Varicella zoster virus infection occurs at a relatively young age in The Netherlands. Vaccine. 2013;31(44):5127–33. doi: 10.1016/j.vaccine.2013.08.029 23973248

[32] Sauerbrei A, Schäfler A, Hofmann J, Schacke M, Gruhn B, Wutzler P. Evaluation of three commercial varicella-zoster virus IgG enzyme-linked immunosorbent assays in comparison to the fluorescent-antibody-to-membrane-antigen test. Clin Vaccine Immunol. 2012;19(8):1261–8. doi: 10.1128/CVI.00183-12 22718131 PMC3416097

[33] Gelman A, Carpenter B. Bayesian Analysis of Tests with Unknown Specificity and Sensitivity. J R Stat Soc Ser C Appl Stat. 2020;69(5):1269–83. doi: 10.1111/rssc.12435 PMC10016948 37252679

[34] Gelman A, Hill J. Data analysis using regression and multilevel/hierarchical models. Cambridge University Press. 2007. doi: 10.1017/CBO9780511790942

[35] Chan DYW, Edmunds WJ, Chan HL, Chan V, Lam YCK, Thomas SL, et al. The changing epidemiology of varicella and herpes zoster in Hong Kong before universal varicella vaccination in 2014. Epidemiol Infect. 2018;146(6):723–34. doi: 10.1017/S0950268818000444 29526171 PMC6533643

[36] Anderson RM, May RM. Infectious diseases of humans: dynamics and control. Oxford University Press. 1991.

[37] Ogunjimi B, Willem L, Beutels P, Hens N. Integrating between-host transmission and within-host immunity to analyze the impact of varicella vaccination on zoster. Elife. 2015;4:e07116. doi: 10.7554/eLife.07116 26259874 PMC4530225

[38] Smits G, Mollema L, Hahné S, de Melker H, Tcherniaeva I, van der Klis F, et al. Seroprevalence of rubella antibodies in The Netherlands after 32 years of high vaccination coverage. Vaccine. 2014;32(16):1890–5. doi: 10.1016/j.vaccine.2014.01.066 24513012

[39] Wiese-Posselt M, Siedler A, Mankertz A, Sauerbrei A, Hengel H, Wichmann O, et al. Varicella-zoster virus seroprevalence in children and adolescents in the pre-varicella vaccine era, Germany. BMC Infect Dis. 2017;17(1):356. doi: 10.1186/s12879-017-2461-2 28525973 PMC5438501

[40] Munasingha H, Amarasinghe A, Malavige N, Sathiakumar N. Seroprevalence of varicella zoster virus in Colombo district, Sri Lanka. Asian Pac J Trop Med. 2018;11(1):53. doi: 10.4103/1995-7645.223561

[41] Akram D, Qureshi H, Mahmud A, Khan AA, Kundi Z, Shafi S. Seroepidemiology of varicella-zoster in Pakistan. The Southeast Asian Journal of Tropical Medicine and Public Health. 2001;31:646–9. 11414405

[42] Meyers J, Logaraj M, Ramraj B, Narasimhan P, MacIntyre CR. Epidemic varicella zoster virus among university students, India. Emerg Infect Dis. 2018;24(2):366–9. doi: 10.3201/eid2402.171427 29350152 PMC5782884

[43] Adam O, Musa A, Kamer A, Benharrat S, Hübschen JM. Active circulation of varicella zoster virus among different age groups in Sudan. Epidemiol Infect. 2022;151:e10. doi: 10.1017/S0950268822001923 36660812 PMC9980927

[44] Mossong J, Putz L, Schneider F. Seroprevalence and force of infection of varicella-zoster virus in Luxembourg. Epidemiol Infect. 2004;132(6):1121–7. doi: 10.1017/s0950268804002754 15635970 PMC2870204

[45] Brisson M, Edmunds WJ, Law B, Gay NJ, Walld R, Brownell M, et al. Epidemiology of varicella zoster virus infection in Canada and the United Kingdom. Epidemiol Infect. 2001;127(2):305–14. doi: 10.1017/s0950268801005921 11693508 PMC2869750

[46] Nardone A, de Ory F, Carton M, Cohen D, van Damme P, Davidkin I, et al. The comparative sero-epidemiology of varicella zoster virus in 11 countries in the European region. Vaccine. 2007;25(45):7866–72. doi: 10.1016/j.vaccine.2007.07.036 17919788

[47] Marin M, Güris D, Chaves SS, Schmid S, Seward JF, Advisory Committee on Immunization Practices, Centers for Disease Control and Prevention (CDC). Prevention of varicella: recommendations of the Advisory Committee on Immunization Practices (ACIP). MMWR Recomm Rep. 2007;56(RR-4):1–40. 17585291

[48] Leung J, McCollum AM, Radford K, Hughes C, Lopez AS, Guagliardo SAJ. Varicella in Tshuapa Province, Democratic Republic of Congo, 2009-2014. Trop Med Int Health. 2019;24(7):839–48. doi: 10.1111/tmi.13248 31062445 PMC8786670

[49] Siddiqi OK, Ghebremichael M, Dang X, Atadzhanov M, Kaonga P, Khoury MN, et al. Molecular diagnosis of central nervous system opportunistic infections in HIV-infected Zambian adults. Clin Infect Dis. 2014;58(12):1771–7. doi: 10.1093/cid/ciu191 24668125 PMC4036687

[50] Hagemann C, Krämer A, Grote V, Liese JG, Streng A. Specific Varicella-Related Complications and Their Decrease in Hospitalized Children after the Introduction of General Varicella Vaccination: Results from a Multicenter Pediatric Hospital Surveillance Study in Bavaria (Germany). Infect Dis Ther. 2019;8(4):597–611. doi: 10.1007/s40121-019-00273-6 31674000 PMC6856245

[51] Hirose M, Gilio AE, Ferronato AE, Ragazzi SLB. The impact of varicella vaccination on varicella-related hospitalization rates: global data review. Rev Paul Pediatr. 2016;34(3):359–66. doi: 10.1016/j.rpped.2015.12.006 26965075 PMC5178123

[52] Varela FH, Pinto LA, Scotta MC. Global impact of varicella vaccination programs. Hum Vaccin Immunother. 2019;15(3):645–57. doi: 10.1080/21645515.2018.1546525 30427766 PMC6605725

[53] Rentier B, Gershon AA, European Working Group on Varicella. Consensus: varicella vaccination of healthy children--a challenge for Europe. Pediatr Infect Dis J. 2004;23(5):379–89. doi: 10.1097/01.inf.0000122606.88429.8f 15131459

[54] Ouwens MJNM, Littlewood KJ, Sauboin C, Téhard B, Denis F, Boëlle P-Y, et al. The impact of 2-dose routine measles, mumps, rubella, and varicella vaccination in France on the epidemiology of varicella and zoster using a dynamic model with an empirical contact matrix. Clin Ther. 2015;37(4):816-829.e10. doi: 10.1016/j.clinthera.2014.12.017 25726457

[55] Marijam A, Safonova E, Scherbakov M, Shpeer E, Van Oorschot D, Rudakova A, et al. Cost effectiveness and budget impact of universal varicella vaccination in Russia. Hum Vaccin Immunother. 2022;18(5):2045152. doi: 10.1080/21645515.2022.2045152 35258445 PMC9196720

[56] Gershon AA, Breuer J, Cohen JI, Cohrs RJ, Gershon MD, Gilden D, et al. Varicella zoster virus infection. Nat Rev Dis Primers. 2015;1:15016. doi: 10.1038/nrdp.2015.16 27188665 PMC5381807

