# Supplementary material for: Trends and patterns of varicella zoster virus infection in Kilifi: A population-based serosurvey
Source: PLOS Glob Public Health. 2026 Aug 18;6(8):e0006013. doi: 10.1371/journal.pgph.0006013 (PMC13484961; doi:10.1371/journal.pgph.0006013)
Supplement: Supplementary file 1 [file pgph.0006013.s008.pdf]

PLOS GLOBAL PUBLIC HEALTH – REVIEWER REPORT

Manuscript ID: PGPH-D-26-00379
Title: *Trends and Patterns of Varicella Zoster Virus Infection in Kilifi: A Population-based Serosurvey*

Summary

This manuscript presents a population-based seroepidemiological analysis of Varicella Zoster Virus (VZV) infection in Kilifi, Kenya, using archived samples collected between 2009 and 2021. The authors estimate age-specific seroprevalence, force of infection (FOI), mean age at infection, and the basic reproduction number (R_0). The findings suggest delayed VZV infection, with substantial susceptibility persisting into adulthood, and propose potential implications for varicella vaccination strategies in Kenya.

The topic is highly relevant to global public health, particularly given the limited data on VZV epidemiology in sub-Saharan Africa.

This manuscript addresses an important gap in VZV epidemiology in sub-Saharan Africa using robust serological data and appropriate modeling approaches. However, there are minor methodological and reporting limitations, that need clarification. I recommend minor revision before the manuscript can be considered for publication. Below is a list of review comments that need addressing.

1. Pooling of heterogeneous serosurveys and potential selection bias. The study combines data from multiple serosurveys conducted for different purposes (malaria, pneumococcal, COVID-19), each likely employing different sampling strategies, risking selection bias. The authors could add a subsection on "harmonization of multiple serosurveys and explicitly describe each dataset. Also need to expand the limitations section to to state the direction of potential bias

2. Insufficient methodological detail for statistical and modeling methods. The manuscripts lacks sufficient detail for reproducibility (likelihood, link function, covariates). The authors should consinder providing full model specifications including likelihood function, distributions and parameters estimated. Also specify the software used and the version numbers. For the catalytic model (FOI Estimation), the authors need to explicitly describe the model fitting approach as well as the model fit metrics

3. The limitations section need to be expanded to sampling bias (non-uniform survey design-data from different surveys), measurement error (Imperfect assay sensitivity/specificity), unmeasured confounding (socioeconomic factors).

4. Reporting – consinder including a STROBE checklist as supplementary file

5. Clarity of terminology - clearly define terms such as population-weighted seroprevalence and bayesian-adjusted estimates for broader readership
