# Supplementary material for: Trends and patterns of varicella zoster virus infection in Kilifi: A population-based serosurvey
Source: PLOS Glob Public Health. 2026 Aug 18;6(8):e0006013. doi: 10.1371/journal.pgph.0006013 (PMC13484961; doi:10.1371/journal.pgph.0006013)
Supplement: Supplementary file 2 [file pgph.0006013.s001.docx]

**S1 Table:** Mid-year population estimates in KHDSS. The average population across the years was used as the standard population.

| Age (years) | 2009 | 2010 | 2011 | 2012 | 2013 | 2014 | 2015 | 2016 | 2017 | 2018 | 2019 | 2020 | 2021 |
| --- | --- | --- | --- | --- | --- | --- | --- | --- | --- | --- | --- | --- | --- |
| 0 | 9620 | 9590 | 10738 | 10191 | 10418 | 10779 | 10372 | 9487 | 9915 | 9285 | 9348 | 7866 | 8225 |
| 1 | 9688 | 9930 | 8835 | 9950 | 9284 | 9186 | 9743 | 9158 | 9092 | 9196 | 9295 | 8878 | 8275 |
| 2 | 9078 | 9769 | 9818 | 8853 | 9975 | 9180 | 8998 | 9398 | 9218 | 9163 | 9187 | 9293 | 9003 |
| 3 | 9447 | 9098 | 9624 | 9787 | 8903 | 9810 | 9057 | 9001 | 9440 | 9128 | 9057 | 8972 | 8985 |
| 4 | 8515 | 9327 | 8997 | 9617 | 9805 | 8849 | 9747 | 9068 | 8930 | 9436 | 9087 | 8812 | 8961 |
| 5 | 8445 | 8448 | 9295 | 9012 | 9709 | 9759 | 8715 | 9650 | 9038 | 8992 | 9478 | 9222 | 8817 |
| 6 | 8703 | 8442 | 8341 | 9272 | 8962 | 9589 | 9652 | 8759 | 9798 | 9119 | 9031 | 9458 | 9005 |
| 7 | 8062 | 8641 | 8442 | 8311 | 9301 | 8998 | 9490 | 9726 | 8871 | 9766 | 9140 | 8939 | 9305 |
| 8 | 8257 | 8000 | 8634 | 8374 | 8348 | 9199 | 8995 | 9635 | 9622 | 8794 | 9759 | 9098 | 8877 |
| 9 | 8153 | 8173 | 7899 | 8514 | 8324 | 8317 | 9153 | 8988 | 9545 | 9735 | 8820 | 9452 | 8985 |
| 10 | 7633 | 8054 | 8120 | 7898 | 8505 | 8297 | 8228 | 9156 | 9014 | 9604 | 9747 | 9151 | 9592 |
| 11 | 7093 | 7665 | 8138 | 8216 | 7900 | 8452 | 8202 | 8210 | 9157 | 9004 | 9564 | 9428 | 8668 |
| 12 | 7212 | 7028 | 7653 | 8139 | 8110 | 7883 | 8368 | 8274 | 8119 | 9177 | 8916 | 9459 | 9560 |
| 13 | 6983 | 7139 | 7043 | 7602 | 8118 | 7946 | 7762 | 8326 | 8131 | 8103 | 9064 | 8942 | 9395 |
| 14 | 6448 | 6863 | 7026 | 6851 | 7331 | 7919 | 7766 | 7557 | 8133 | 8005 | 7833 | 8703 | 8592 |
