# Supplementary material for: Trends and patterns of varicella zoster virus infection in Kilifi: A population-based serosurvey
Source: PLOS Glob Public Health. 2026 Aug 18;6(8):e0006013. doi: 10.1371/journal.pgph.0006013 (PMC13484961; doi:10.1371/journal.pgph.0006013)
Supplement: Supplementary file 3 [file pgph.0006013.s002.docx]

**S2 Table:** Comparison of seroprevalence of anti-VZV IgG antibody among children aged 0-14 years across all years.

| Surveys | Year | All  N (%) | Crude seroprevalence  95% CI | Bayesian-adjusted seroprevalence  95% CI |
| --- | --- | --- | --- | --- |
| Malaria cross-sectional | 2009 | 363 (13.6) | 31.4 (26.8-36.5) | 38.5 (29.0-46.9) |
|  | 2011 | 304 (11.4) | 46.4 (40.7-52.2) | 45.6 (38.2-53.1) |
|  | 2013 | 407 (15.2) | 41.5 (36.7-46.5) | 39.5 (33.2-46.3) |
|  |  |  |  |  |
| Pneumococcal biennial serosurvey | 2015 | 373 (14.0) | 25.7 (21.4-30.5) | 29.9 (21.8-37.4) |
|  | 2017 | 417 (15.7) | 24.0 (20.0-28.4) | 28.3 (22.0-35.4) |
|  | 2019 | 516 (19.4) | 27.9 (24.1-32.0) | 33.9 (26.6-40.5) |
|  |  |  |  |  |
| COVID-19 serosurvey | 2021 | 284(10.7) | 46.5 (40.6-52.5) | 43.8 (36.2-52.0) |
|  |  | 2662 (100) |  |  |
