# Supplementary material for: Trends and patterns of varicella zoster virus infection in Kilifi: A population-based serosurvey
Source: PLOS Glob Public Health. 2026 Aug 18;6(8):e0006013. doi: 10.1371/journal.pgph.0006013 (PMC13484961; doi:10.1371/journal.pgph.0006013)
Supplement: Supplementary file 4 [file pgph.0006013.s007.docx]

**Supplementary S4:** STROBE Checklist (Strengthening the Reporting of Observational Studies in Epidemiology). Reproduced under the terms of the Creative Commons Attribution 4.0 International (CC BY 4.0) License.

|  | Item No | Recommendation |
| --- | --- | --- |
| **Title and abstract** | 1 | Cross-section population-based is stated in the abstract (page 2). |
|  |  | An informative and balanced summary of what was done and what was found has been provided in the abstract section in (Abstract, page 2). |
| Introduction | | |
| Background/rationale | 2 | The introduction (pages 4-5) highlights existing population-based serological studies on varicella zoster virus (VZV) from high-income countries and the lack of such data in Kenya as the rationale for the investigation being reported. |
| Objectives | 3 | The overall study objective was to determine age-specific immunity profiles for VZV in healthy children and adults and associated risk factors (Introduction, page 5). |
| Methods | | |
| Study design | 4 | The description of the pooled cross-sectional surveys (malaria cross-sectional survey, pneumococcal vaccine impact (PCVIS) cross-sectional surveys, and COVID-19 serosurveys) have been described in detail (Materials and Methods, page 6) |
| Setting | 5 | The primary surveys were conducted in Kilifi Health Demographic Surveillance System (KHDSS) between 2009 and May 2021. The reference to the details the geographic cover of the KHDSS and the demographic of the population has been provided (Materials and Methods, page 6). |
| Participants | 6 | Random selection of 2355 children aged 0-15 years were recruited into the malaria and PCVIS survey while COVID-19 serosurvey obtained a random sample of 830 participants across all age groups in an age-stratified random sample (Materials and Methods, pages 6-7). |
| Variables | 7 | The primary outcomes was ant-VZV IgG seropositivity using seropositivity threshold of ≥0.26 IU/mL. The confounding variables included age category, survey year, and child’ sex (Materials and Methods, page 8-9). |
| Data sources/ measurement | 8* | The primary variable-VZV immunoglobulin G (IgGs) was measured using fluorescent-bead-based multiplex immunoassay on the Luminex® MAGPIX® (Materials and Methods, page 7). Metadata of the participants was obtained from the study database from each of the three primary surveys (Materials and Methods, pages 7-8). |
| Bias | 9 | We computed population-weighted VZV seroprevalence using KHDSS population structure to account for differences in the sampling in the three survey to minimize sampling bias (Materials and Methods, pages 9) |
| Study size | 10 | The sample size of each of the pooled surveys was estimated to provide sufficient precision for age-specific VZV seroprevalence with approximately 50 participants in each age groups (Materials and Methods, pages 6). |
| Quantitative variables | 11 | The participants’ age was analysed as categorical and continuous variables. The VZV IgGs seropositivity was analysed as proportions (prevalence) and as log transformed IgGs continuous variable. |
| Statistical methods | 12 | We calculated crude VZV seroprevalence stratified by age, survey type, and sex. We then computed population-weighted seroprevalence of anti-VZV IgG using average population structure of KHDSS (Materials and Methods, page 9-10). |
|  |  | Bayesian hierarchical logistic regression model with logit link function was fitted to explore factors associated with VZV seropositivity. We then fitted catalytic model using age-specific seroprevalence data to estimate age-specific force of infection in the susceptible population. Posterior distributions were estimated using the Markov Chain Monte Carlo (MCMC) methods (Materials and Methods, page 9-11). |
|  |  | Samples missing metadata of varicella serological data were dropped from analyses. |
|  |  | VZV IgG seroprevalence estimates were standardized to the annual KHDSS population age distribution for each survey year and adjusted for assay sensitivity and specificity to improve population representativeness and account for potential serostatus misclassification arising from imperfect assay performance (Materials and Methods, page 8-11). |
|  |  | Sensitivity analyses was done to assess the impact of wanning immunity following natural varicella infection (Materials and Methods, page 9-10). |
| Results | | |
| Participants | 13* | The study analysed samples from 2639 children aged 0-14 years and 546 children and adults aged ≥15. A summary of the sociodemographic characteristics has been provided in table 1 (Results, pages 13-14). |
|  |  | Samples with missing metadata were dropped from analysis |
|  |  | Table 1 gives summary of the participants distribution by sex across survey years (Results, pages 13-14). |
| Descriptive data | 14* | The characteristics of study participants e.g. demographics and information on VZV exposure and potential confounders have been provided. Table 1 (Results, pages 13-14) and table 2 (Results, page 16). |
|  |  | Samples with missing metadata were dropped from analysis |
| Outcome data | 15* | Bayesian age-standardised seroprevalence of VZV IgG antibodies for children aged 0-14 ranged from 28.3% (95% CI 22.0-35.4) in 2017 to 45.6% (95% CI 38.2-53.1) in 2011 (Results, page 13). |
| Main results | 16 | Seroprevalence of VZV IgG antibodies was consistently lowest among children aged 0-4 years, ranging from 5.5% (95% CI 1.0-11.4) in 2017 to 25.3 % (95% CI 14.5-36.8) in 2021. In contrast, seroprevalence among children aged 5-9 years ranged from 29.7% (95% CI 20.7-38.6) in 2017 to 55.2% (95% CI 43.1-66.4) in 2021 (Results, page 14-15). These findings were adjusted for survey year and age category to assess whether VZV seropositivity was associated with years of the study or age (Results, pages 15-17). |
|  |  | Age groups for the participants were stratified as: 0-4, 5-9, 10-14, 15-24, 25-34, 35-44, 45-54, 55-64, and ≥65. The outcome variable was VZV serostatus, defined as a binary variable using anti-VZV IgG cutoff of 0.26 IU/mL (Results, pages 16-17) |
| Other analyses | 17 | The highest force of infection was in age 5-9 years with corresponding reproduction number of 2.54 and herd immunity threshold was 61% (Results, page 17). |
| Discussion | | |
| Key results | 18 | The study showed that about 38% of children aged 0-14 had serological evidence of prior VZV infection and this increased to >80% by age 45 years. The highest rate of infection was observed in children aged 5-9. Age was the strongest predictor of VZV seropositivity (page 18-19). |
| Limitations | 19 | The limitations in this study include variability of primary surveys designs, predominantly rural population, and imperfect assay. This might have introduced bias to anti-VZV IgGs seroprevalence (Discussion, page 21-22). |
| Interpretation | 20 | This study shows an increase in anti-VZV IgG seropositivity with age, the FOI is low resulting in about 40% of children transitioning into adulthood without VZV immunity. The observed transmission pattern across all ages reflected continued infection into adulthood, suggesting a hidden burden of severe VZV disease among adolescents and adults including sequelae such as congenital varicella syndrome (Discussion, page 22-23). |
| Generalisability | 21 | The findings of this study may be generalised to similar rural settings in Kenya (Discussion, page 22) |
| Other information | | |
| Funding | 22 | Funding statement provided; funders had no role in study design, analysis, or publication decisions. |
