# Supplementary material for: Trends and patterns of varicella zoster virus infection in Kilifi: A population-based serosurvey
Source: PLOS Glob Public Health. 2026 Aug 18;6(8):e0006013. doi: 10.1371/journal.pgph.0006013 (PMC13484961; doi:10.1371/journal.pgph.0006013)
Supplement: Supplementary file 5 [file pgph.0006013.s009.docx]

This manuscript examines the seroepidemiology of VZV in Kilifi, Kenya where varicella vaccine is not currently included in the national infant immunization schedule. The study is valuable given the relative scarcity of population-based VZV seroprevalence data from LMICs, despite their importance for understanding disease burden and informing immunization policy decisions. By estimating key epidemiological parameters, the findings may also contribute to understanding transmission dynamics and informing future transmission models, particularly for LMIC settings. Strengths of the study include its population-based seroprevalence estimates derived from large random samples across multiple age groups, as well as the application of catalytic models to estimate key epidemiological parameters from retrospective cross-sectional data. My main concerns relate to the clarity and interpretation of the methodological framework and results presentation; several analytical methods and epidemiological concepts would benefit from additional explanation and context for the readership of PLOS Global Health. The manuscript would also benefit from greater clarity and consistency in the presentation of results and figures, as well as expanded discussion of the study limitations and their implications for generalizability. Overall, this is a valuable study with important contribution, but requires some clarification and improved interpretation/reporting.

Major comments:

1. *Clarification of Bayesian estimation framework (lines 167-170)*: Consider providing additional context for the Bayesian estimation procedures for readers less familiar with these methods. Briefly distinguish MCMC as the general framework from Gibbs sampling as the specific algorithm used and clarify why this approach was applied for parameter estimation. Please also relate the Gelman-Rubin statistic to the overall framework (e.g., “convergence of MCMC chains…”).
2. *Interpretation of R0 and its relationship to mean age at infection (lines 171-186)*: Please define R0 in epidemiological terms and provide additional interpretive context for the relationship R0 = L/A (e.g., younger mean age at infection corresponds to higher transmission intensity and larger R0).
3. *HIT and translation to vaccination coverage:* The HIT is calculated from R0 estimated based on natural infection. In this context, it’s reasonable that primary vaccine failure is not included. It would be helpful to note in the discussion, though, that this theoretical threshold does not directly translate to vaccination coverage targets, and that vaccine efficacy should be considered when determining the coverage needed to achieve herd immunity.
4. *Clarity and consistency of IgG concentrations and figures* (Figure 1B, S1A and S1B):
   a. Lines 216-222: There appears to be a lack of clarity regarding whether VZV IgG concentrations are reported on the original or log-transformed scale. The text describes “log-transformed VZV IgG antibody” but reports values in IU/mL that appear to correspond to back-transformed geometric mean concentrations, while Figure 1B appears to display log-transformed values (including negative means). Please clarify the scale used for both the statistical summaries and the figure to ensure consistency in interpretation. In addition, the numerical values reported in the text should be checked for consistency with those shown in Figure 1B, as there appear to be minor discrepancies. Also in line 220, “lowest” of what? E.g., Please correct to “lowest concentration of 0.04…”
   b. Lines 236-238: Please clarify if these values are averaged across years or referencing a specific year.
   c. Lines 242-244: Please describe the pattern (e.g., above the seropositivity threshold).
5. *Generalizability to other settings* (line 368): The study was conducted in a rural coastal setting, which as mentioned in the limitations section, may limit generalizability. Given that varicella transmission is strongly dependent on contact patterns and age structure, it would be helpful if the authors could discuss how FOI, R), or other estimates might differ in urban or higher density settings.

Minor comments:

- Lines 49-52 are missing citations
- Some in-text citations are missing a space between the text and citation. Please correct. Examples include line 69 and 71.
- Please correct “95%CI” to “95% CI” where it appears throughout the manuscript and in the tables.
- Line 125: a space is needed between “post-stratification” and “(MLRP)”
- Lines 130-131: Please cite the appropriate methodological references (rather than a prior applied study) and provide sufficient detail on the specification of the non-informative priors used (e.g., distributions and variance/hyperparameters) to ensure reproducibility.
- Line 185: Please center-align the equation.
- Table 1: Please add the statistical test conducted (e.g., in footnotes)
- Line 215: Incomplete sentence. Please correct.
- Table 2: For age groups 15 years and over, please either remove or clarify the value that is in parentheses. For example, for age group 15-24, N = 105 (3.3). What is 3.3?
- Figures 3 and 4: Please modify captions to be more descriptive: “…the associated binomial 95% confidence intervals **by the black vertical lines**”. I assume the gray band around the solid horizontal line (model predictions) is the 95% CI, but please clarify this in the caption.
- Line 268: Please modify to “This corresponds to a decrease in **the** reproduction number…”
- Line 270: Please specify “The **median** FOI…”
- Table 3: In the footnote, the terms “first” and “second” catalytic model may be ambiguous. Is this in reference to chronology or to model structure/specifications? Please clarify.
- Lines 293-295: The waning rate is reported as 0.087 per year, but its interpretation is not entirely clear. Please clarify the epidemiological meaning of this parameter: is it the annual rate or hazard of losing immunity, seropositivity, or detectable antibodies?
- Line 328: “Available evidence show” should be “Available evidence shows”
- Please review in-text references to figures and tables and ensure that they are consistent in their typography according to PLOS formatting style. For example, in line 128, Table S2 is unnecessarily bolded. In line 264, the “f” in “(figure 3)…(figure 4)” should be capitalized. And in line 272, “(*Table 3*)” is unnecessarily bolded and italicized.
