# Supplementary material for: Trends and patterns of varicella zoster virus infection in Kilifi: A population-based serosurvey
Source: PLOS Glob Public Health. 2026 Aug 18;6(8):e0006013. doi: 10.1371/journal.pgph.0006013 (PMC13484961; doi:10.1371/journal.pgph.0006013)
Supplement: Supplementary file 6 [file pgph.0006013.s011.docx]

Antipa Kirwa Sigilai,

Research Fellow, Institute for Human Development ,

Aga Khan university.

PO Box 30270-00100, Nairobi, Kenya

Email: antipa.sigilai@aku.edu

Date: 24^th^ June 2026

Editor-in-Chief,

PLOS Global Public Health.

Dear Editor,

Thank you for your constructive and insightful comments provided which have helped us to improve the clarity, rigor, and overall quality of our manuscript. We appreciate the expertise and the time invested by the reviewers and have satisfactorily addressed all concerns.

Please see a point-by-point response below.

**Reviewer 1**

*This manuscript examines the seroepidemiology of VZV in Kilifi, Kenya where varicella vaccine is not currently included in the national infant immunization schedule. The study is valuable given the relative scarcity of population-based VZV seroprevalence data from LMICs, despite their importance for understanding disease burden and informing immunization policy decisions. By estimating key epidemiological parameters, the findings may also contribute to understanding transmission dynamics and informing future transmission models, particularly for LMIC settings. Strengths of the study include its population-based seroprevalence estimates derived from large random samples across multiple age groups, as well as the application of catalytic models to estimate key epidemiological parameters from retrospective cross-sectional data. My main concerns relate to the clarity and interpretation of the methodological framework and results presentation; several analytical methods and epidemiological concepts would benefit from additional explanation and context for the readership of PLOS Global Health. The manuscript would also benefit from greater clarity and consistency in the presentation of results and figures, as well as expanded discussion of the study limitations and their implications for generalizability. Overall, this is a valuable study with important contributions, but requires some clarification and improved interpretation/reporting.*

**Response:**

We thank the reviewer for the positive and constructive feedback.

***Major comment 1:***

*Clarification of Bayesian estimation framework (lines 167-170): Consider providing additional context for the Bayesian estimation procedures for readers less familiar with these methods. Briefly distinguish MCMC as the general framework from Gibbs sampling as the specific algorithm used and clarify why this approach was applied for parameter estimation. Please also relate the Gelman-Rubin statistic to the overall framework (e.g., “convergence of MCMC chains…”).*

**Response:**

We have expanded the description of the Bayesian estimation procedure to clarify that Markov chain Monte Carlo (MCMC) is the general framework used for posterior inference, while Gibbs sampling is the specific algorithm implemented in rjags for posterior sampling. We have also clarified that convergence was assessed using the Gelman–Rubin diagnostic applied to multiple MCMC chains and have provided additional details on the prior distributions, number of chains, adaptation period, and sampling iterations (**line** **190-199**).

***Major comment 2:***

*Interpretation of R0 and its relationship to mean age at infection (lines 171-186)*: Please define R0 in epidemiological terms and provide additional interpretive context for the relationship R0 = L/A (e.g., younger mean age at infection corresponds to higher transmission intensity and larger R0).

**Response:**

We have defined the basic reproduction number (R₀) as the average number of secondary infections generated by a typical infectious individual in a fully susceptible population. We have also added explanatory text describing the inverse relationship between R₀ and the mean age at infection, noting that higher transmission intensity results in earlier infection and therefore a lower mean age at infection and higher R₀ (**line** **209-219**). Additional interpretation linking the estimated mean age at infection and R₀ to delayed acquisition of varicella in Kilifi has also been added to the Discussion (**line** **337-339**).

***Major comment 3:***

*HIT and translation to vaccination coverage:* The HIT is calculated from R0 estimated based on natural infection. In this context, it’s reasonable that primary vaccine failure is not included. It would be helpful to note in the discussion, though, that this theoretical threshold does not directly translate to vaccination coverage targets, and that vaccine efficacy should be considered when determining the coverage needed to achieve herd immunity.

**Response:**

We agree that the herd immunity threshold estimated from R₀ represents a theoretical transmission threshold and does not directly translate into a vaccination coverage target. We have therefore added text to the Discussion noting that the coverage required to achieve herd immunity would depend on vaccine effectiveness, duration of protection, and heterogeneity in contact patterns, and may differ from the theoretical threshold estimated under assumptions of homogeneous transmission (**line** **369-374**)

***Major comment 4a:***

a) *Clarity and consistency of IgG concentrations and figures (Figure 1B, S1A and S1B):*

*Lines 216-222: There appears to be a lack of clarity regarding whether VZV IgG concentrations are reported on the original or log-transformed scale. The text describes “log-transformed VZV IgG antibody” but reports values in IU/mL that appear to correspond to back-transformed geometric mean concentrations, while Figure 1B appears to display log-transformed values (including negative means). Please clarify the scale used for both the statistical summaries and the figure to ensure consistency in interpretation. In addition, the numerical values reported in the text should be checked for consistency with those shown in Figure 1B, as there appear to be minor discrepancies. Also in line 220, “lowest” of what? E.g., Please correct to “lowest concentration of 0.04…”*

**Response:**

The GMC estimates reported in the text were calculated on the original IU/mL scale and represent back-transformed summaries, whereas Figure 1B displays the distribution of individual log-transformed VZV IgG concentrations. We have revised the text and figure caption to clarify the scale used for each summary measure and to improve consistency in interpretation. We have also corrected the wording describing the minimum GMC observed in 2017 (**line** **247-259**)

***Major comment 4b:***

*b) Lines 236-238: Please clarify if these values are averaged across years or referencing a specific year.*

**Response:**

We have clarified this as the mean GMCs concentration across all survey years **(Line 274-275)**.

***Major comment 4c:***

*c) Lines 242-244: Please describe the pattern (e.g., above the seropositivity threshold).*

**Response:**

We have revised this to show that the figures were above the seropositivity threshold (**Line 279-281**).

**Major comment 5:**

*Generalizability to other settings (line 368): The study was conducted in a rural coastal setting, which as mentioned in the limitations section, may limit generalizability. Given that varicella transmission is strongly dependent on contact patterns and age structure, it would be helpful if the authors could discuss how FOI, R), or other estimates might differ in urban or higher density settings.*

**Response:**

We have expanded the limitations section to discuss the potential impact of setting-specific differences in contact patterns, household structure, school mixing, and population density on VZV transmission. We now note that transmission intensity and force of infection may differ in urban or other higher-density settings and that this could lead to earlier ages at infection and different transmission dynamics than those observed in rural coastal Kenya (**Lines 401-405**).

***Minor comment 1:***

*Lines 49-52 are missing citations*

**Response:**

We have added the WHO 2014 position paper on varicella zoster virus as the reference (**Line 50-53**).

***Minor comment 2:***

*Some in-text citations are missing a space between the text and citation. Please correct. Examples include line 69 and 71.*

**Response:**

The space has been added as suggested **(Line 69-70)**

***Minor comment 3:***

*Please correct “95%CI” to “95% CI” where it appears throughout the manuscript and in the tables*.

**Response:**

We have corrected this in the entire manuscript where “95%CI” has been changed to “95% CI”

***Minor comment 4:***

Line 125: a space is needed between “post-stratification” and “(MLRP)”

**Response:**

We have revised this accordingly **(line 139)**.

***Minor comment 4:***

Lines 130-131: Please cite the appropriate methodological references (rather than a prior applied study) and provide sufficient detail on the specification of the non-informative priors used (e.g., distributions and variance/hyperparameters) to ensure reproducibility.

**Response**

We have expanded the description of the MRP methodology and added methodological references describing multilevel regression and post-stratification and Bayesian adjustment for imperfect assay sensitivity and specificity. We now provide details of the model structure, including the binomial likelihood, hierarchical age effects, incorporation of assay performance data, and the prior distributions used in the Bayesian model (**line 129-143**).

***Minor comment 4:***

*Line 185: Please center-align the equation.*

**Response:**

We have centre-aligned the equation (**line 220**)

***Minor comment 5:***

*Table 1: Please add the statistical test conducted (e.g., in footnotes)*

**Response:**

We have added statistical test conducted on the footnote **(Line 244-245)**

***Minor comment 6:***

*Line 215: Incomplete sentence. Please correct.*

**Response:**

We have revised this to make it more succinct **(line 247-250)**

***Minor comment 7:***

Table 2: For age groups 15 years and over, please either remove or clarify the value that is in parentheses. For example, for age group 15-24, N = 105 (3.3). What is 3.3?

**Response**

We have removed the values in parenthesis. This was the proportion of each age category but upon review we acknowledge it does not add much value.

***Minor comment 7:***

*Figures 3 and 4: Please modify captions to be more descriptive: “…the associated binomial 95% confidence intervals* ***by the black vertical lines****”. I assume the gray band around the solid horizontal line (model predictions) is the 95% CI, but please clarify this in the caption.*

**Response**

We have revised the captions of Figures 3 and 4 to more clearly describe the figures, including the observed seroprevalence estimates (black dots), binomial 95% confidence intervals (black vertical lines), median model predictions (solid line), and the 95% credible intervals around the model predictions (grey shaded region).

***Minor comment 8:***

*Line 268: Please modify to “This corresponds to a decrease in* ***the*** *reproduction number…”*

**Response:**

We have now revised this **(line 307)**

***Minor comment 9:***

*Line 270: Please specify “The* ***median*** *FOI…”*

**Response:**

We have now revised this **(line 309-310)**

***Minor comment 10:***

*Table 3: In the footnote, the terms “first” and “second” catalytic model may be ambiguous. Is this in reference to chronology or to model structure/specifications? Please clarify.*

**Response:**

We have revised the footnote to explicitly describe the populations and datasets used for each catalytic model (**Line 318-320**)

***Minor comment 11:***

*Lines 293-295: The waning rate is reported as 0.087 per year, but its interpretation is not entirely clear. Please clarify the epidemiological meaning of this parameter: is it the annual rate or hazard of losing immunity, seropositivity, or detectable antibodies?*

**Response:**

We have clarified that the waning parameter in the reversible catalytic model represents the annual rate of transition from the seropositive to the seronegative state and should therefore be interpreted as a model-based estimate of loss of seropositivity .This clarification has been added to the Results section (**line 333**)

***Minor comment 12:***

Line 328: “Available evidence show” should be “Available evidence shows”

**Response:**

We have corrected this (**line 378**)

***Minor comment 13:***

Please review in-text references to figures and tables and ensure that they are consistent in their typography according to PLOS formatting style. For example, in line 128, Table S2 is unnecessarily bolded. In line 264, the “f” in “(figure 3)…(figure 4)” should be capitalized. And in line 272, “(*Table 3*)” is unnecessarily bolded and italicized.

**Response:**

We have corrected all tables and figures in-text referencing across all the manuscript in accordance with PLOS formatting style. For figures (Lines 250, 257, 273, 281, 282, 302, and 337). For tables (Lines 142, 241, 251, 296, 310, and 337).

**Reviewer 2**

*This manuscript presents a population-based seroepidemiological analysis of Varicella Zoster Virus (VZV) infection in Kilifi, Kenya, using archived samples collected between 2009 and 2021. The authors estimate age-specific seroprevalence, force of infection (FOI), mean age at infection, and the basic reproduction number (R₀). The findings suggest delayed VZV infection, with substantial susceptibility persisting into adulthood, and propose potential implications for varicella vaccination strategies in Kenya.The topic is highly relevant to global public health, particularly given the limited data on VZV epidemiology in sub-Saharan Africa.This manuscript addresses an important gap in VZV epidemiology in sub-Saharan Africa using robust serological data and appropriate modeling approaches. However, there are minor methodological and reporting limitations that need clarification. I recommend minor revision before the manuscript can be considered for publication. Below is a list of review comments that need addressing.*

**Response:**

We thank the reviewer for the positive and constructive feedback.

***Comment 1***

*Pooling of heterogeneous serosurveys and potential selection bias. The study combines data from multiple serosurveys conducted for different purposes (malaria, pneumococcal, COVID-19), each likely employing different sampling strategies, risking selection bias. The authors could add a subsection on “harmonization of multiple serosurveys and explicitly describe each dataset. Also need to expand the limitations section to to state the direction of potential bias*

**Response:**

We have added a subsection describing the harmonization **(Line 103-116)** of the multiple serosurveys and now explicitly describe the source datasets, their original objectives, and the common population-based sampling framework within the Kilifi Health and Demographic Surveillance System. We have also expanded the limitations section to discuss the potential for residual selection bias arising from pooling surveys conducted for different purposes. Because participant selection was independent of VZV infection status, the direction of any resulting bias is uncertain and could potentially lead to either over- or under-estimation of seroprevalence if sampled individuals differed systematically from the underlying population in their cumulative exposure to VZV **(Line 454-461)**

***Comment 2***

*Insufficient methodological detail for statistical and modeling methods. The manuscripts lacks sufficient detail for reproducibility (likelihood, link function, covariates). The authors should consider providing full model specifications including likelihood function, distributions and parameters estimated. Also specify the software used and the version numbers. For the catalytic model (FOI Estimation), the authors need to explicitly describe the model fitting approach as well as the model fit metrics*

**Response:**

We thank the reviewer for this suggestion. We have expanded the description of both the Bayesian MRP and catalytic modelling approaches to improve reproducibility. For the MRP analysis, we now provide details of the model structure, including the binomial likelihood, logit link function, hierarchical age effects, incorporation of assay sensitivity and specificity, prior distributions, software implementation, and the post-stratification procedure used to generate age- and test-adjusted seroprevalence estimates **(lines 129-143)**. For the catalytic model, we have expanded the description of the fitting procedure, including the likelihood function, prior distributions, age-specific force of infection parameters estimated, MCMC implementation, convergence assessment using trace plots and the Gelman-Rubin diagnostic (R̂ < 1.01), and evaluation of model fit through visual comparison of observed and model-predicted age-specific seroprevalence estimates **(lines 166-174)**.

***Comment 3***

*The limitations section needs to be expanded to sampling bias (non-uniform survey design data from different surveys), measurement error (Imperfect assay sensitivity/specificity), unmeasured confounding (socioeconomic factors).*

**Response:**

We have captured these limitations. See **lines 427- 431** responds to comment on (non-uniform survey design data from different surveys) and unmeasured socioeconomic factors **(lines 434-436)**

***Comment 4***

*Reporting – consider including a STROBE checklist as supplementary file*

**Response:**

This has been added as a supplementary material and uploaded separately as appropriate **(Supplementary S4).**

***Comment 5***

*Clarity of terminology - clearly define terms such as population-weighted seroprevalence and bayesian-adjusted estimates for broader readership*

**Response:**

We have clarified both terms at first use in the Methods. Specifically, we now explain that Bayesian-adjusted seroprevalence estimates were obtained from a Bayesian model accounting for uncertainty in assay sensitivity and specificity, and that population-weighted seroprevalence estimates were calculated by weighting age-specific estimates according to the demographic structure of the study population.

We hope that this revision satisfactorily address all comments and concerns raised by the reviewers.

Sincerely,

Corresponding Author: Antipa Sigilai
