# Supplementary material for: Trends and patterns of varicella zoster virus infection in Kilifi: A population-based serosurvey
Source: PLOS Glob Public Health. 2026 Aug 18;6(8):e0006013. doi: 10.1371/journal.pgph.0006013 (PMC13484961; doi:10.1371/journal.pgph.0006013)
Supplement: Supplementary file 7 [file pgph.0006013.s012.docx]

Antipa Kirwa Sigilai,

Research Fellow, Institute for Human Development ,

Aga Khan University.

PO Box 30270-00100, Nairobi, Kenya

Email: antipa.sigilai@aku.edu

Date: 9^th^ July 2026

Editor-in-Chief,

PLOS Global Public Health.

Dear Editor,

Thank you for your careful evaluation of our improved manuscript and the constructive minor comments that you have raised. We appreciate the expertise and the time invested by the reviewers and have satisfactorily addressed all concerns.

Please see a point-by-point response below.

**Comment**  **1**
1. Please revise the conclusion to avoid implying that this study directly measured adult VZV disease burden or demonstrated vaccine impact. The current conclusion states that the data show “a significant VZV burden in adults which can be prevented by a childhood vaccine programme.” This wording should be tempered because the study measured VZV IgG seroprevalence, susceptibility, and modelled force of infection, but did not directly measure clinical disease burden, complications, hospitalizations, mortality, congenital varicella syndrome, or vaccine effectiveness.
Please revise this language so that the conclusion is proportionate to the data presented. For example, instead of:
“Taken together, seroepidemiological and modelling data show a significant VZV burden in adults which can be prevented by a childhood vaccine programme.”
Please consider revising to:
“Taken together, the seroepidemiological and modelling data identify a substantial susceptibility gap extending into adolescence and adulthood, suggesting the potential for continued VZV infection at older ages. These findings support the need for additional studies of VZV morbidity burden, vaccine impact, cost-effectiveness, and programmatic feasibility to inform varicella vaccine policy in Kenya.”

**Response**

We have clarified this conclusion as suggested in **pages 22-23, lines 443-448**

**Comment 2**

2. Please ensure that the final submitted manuscript is fully clean, with all tracked-change artifacts, duplicated text, editorial comments, formatting remnants, and inconsistent figure/table references removed. For example, the sentence explaining the relationship between R₀ and mean age at infection currently appears to contain a corrupted phrase: “populations experiencing higher transvalues. Uncertainty tend…” Please revise this sentence so that it reads clearly and grammatically.

**Response**

We have revised these two sentences to make them succinct**.** Revised sentences are in **page 12, lines 217-221**

**Comment 2**
3. Please revise the figures to improve readability and ensure they meet publication-quality standards.
For Figure 1, please ensure that all survey years are fully visible on the x-axis and that the axis labels, panel labels, and seropositivity threshold line are legible at final publication size. Please also confirm that the caption clearly distinguishes between the GMC estimates reported in the text on the original IU/mL scale and the log-transformed IgG concentrations shown in panel B.
For Figure 2, please improve the spacing of the faceted panels and x-axis age-category labels. In the current version, the age-category labels are crowded, and the rightmost panel appears difficult to read. Please ensure that each survey year panel is fully visible, that the age categories are not clipped or overlapping, and that the seropositivity threshold line is clearly labelled or explained in the caption.
For Figure 3, please ensure that all panel labels, age-axis labels, observed points, confidence intervals, model-predicted line, and credible interval shading are legible. Please also confirm that the figure caption clearly states what the black points, black vertical lines, solid line, and grey shaded area represent.
For Figure 4, please consider simplifying the presentation of the adult seroprevalence model because the current figure is visually crowded, with many points and wide confidence intervals. One option would be to present age-banded estimates rather than many individual-age estimates. Another option would be to move the current detailed figure to the supplement and include a clearer main-text figure showing seroprevalence by adult age band with model estimates and uncertainty. At minimum, please improve the scaling and spacing so the data points, uncertainty intervals, model line, and grey credible interval band can be interpreted clearly.

**Response**

**Figure 1:** We have reworked on the figures 1-3 as suggested with visibility of all survey years and we improved x-axis panels and labels. We have also made the caption succinct to distinguish between GMC estimates reported and log-transformed concentration in panel B. **Page 14, lines 262-269**

**Figure 2:** We have improved spacing of the faceted panel and x-axis age-category labels. We have also ensured visibility of each survey year as suggested by the editors. Threshold line has also been explained in the caption**. Page 15, lines 288-294.**

**Figure 3:** We have made panel labels on age-axis eligible. We have also revised caption to make it clear with explanations of the black points, black vertical lines, solid line, and grey shaded area. **Page 17, lines 326-331**

**Figure 4:** We have simplified this figure and moved it to supplementary section as **figure S3**. **Page 32, lines 653-657**

**Comment 4**
4. Please review the data availability statement and ensure that it clearly explains the process for accessing the underlying data. If the data cannot be deposited publicly because of ethical, legal, or governance restrictions, please state this explicitly and provide sufficient detail on how qualified researchers may request access.

**Response**

I have revised data availability statement to ensure it explains the process of accessing data.

We hope that this revision addresses all comments raised by the reviewers.

Sincerely,

Corresponding Author: Antipa Sigilai
