# Supplementary material for: Trends and patterns of varicella zoster virus infection in Kilifi: A population-based serosurvey
Source: PLOS Glob Public Health. 2026 Aug 18;6(8):e0006013. doi: 10.1371/journal.pgph.0006013 (PMC13484961; doi:10.1371/journal.pgph.0006013)
Supplement: Peer review history

# Peer review: Decision Letter 0

**DOI:** 10.1371/journal.pgph.0006013.r001

**Authors:** Maria del Pilar Fernandez

10 May 2026

PGPH-D-26-00379

Trends and Patterns of Varicella Zoster Virus Infection in Kilifi: A Population-based Serosurvey

PLOS Global Public Health

Dear Dr. Sigilai,

Thank you for submitting your manuscript to PLOS Global Public Health. After careful consideration, we feel that it has merit but does not fully meet PLOS Global Public Health’s publication criteria as it currently stands. Therefore, we invite you to submit a revised version of the manuscript that addresses the points raised during the review process.

==============================

For further consideration, the authors must clarify the analytical and modeling framework; ensure consistency between the text, tables, and figures; clarify the interpretation of R0, FOI, HIT, and antibody waning; address the Data Availability Statement in line with PLOS policy; and expand the limitations regarding generalizability beyond the rural coastal study setting.

Other reviewer comments related to wording, formatting, citations, and table/figure presentation should also be addressed, but these are recommended unless they affect interpretation or reproducibility.

There is no substantive conflict between the reviews. Although the reviewers differed in the extent of revision recommended, both identified issues that require clarification before acceptance. Given the scope of these revisions, a major revision decision is appropriate.

==============================

Please submit your revised manuscript by Jul 09 2026 11:59PM. If you will need more time than this to complete your revisions, please reply to this message or contact the journal office at globalpubhealth@plos.org. When you're ready to submit your revision, log on to https://www.editorialmanager.com/pgph/ and select the 'Submissions Needing Revision' folder to locate your manuscript file.

Please include the following items when submitting your revised manuscript:

- A letter that responds to each point raised by the editor and reviewer(s). You should upload this letter as a separate file labeled 'Response to Reviewers'.
- A marked-up copy of your manuscript that highlights changes made to the original version. You should upload this as a separate file labeled 'Revised Manuscript with Track Changes'.
- An unmarked version of your revised paper without tracked changes. You should upload this as a separate file labeled 'Manuscript'.

Guidelines for resubmitting your figure files are available below the reviewer comments at the end of this letter.

As the corresponding author, your ORCID iD is verified in the submission system and will appear in the published article. PLOS supports the use of ORCID, and we encourage all coauthors to register for an ORCID iD and use it as well. Please encourage your coauthors to verify their ORCID iD within the submission system before final acceptance, as unverified ORCID iDs will not appear in the published article. *Only*  the individual author can complete the verification step; PLOS staff *cannot*  verify ORCID iDs on behalf of authors.

We look forward to receiving your revised manuscript.

Kind regards,

Maria del Pilar Fernandez

Academic Editor

PLOS Global Public Health

**Journal Requirements:**

1.  Please amend your detailed Financial Disclosure statement. This is published with the article. It must therefore be completed in full sentences and contain the exact wording you wish to be published.

i. Please clarify all sources of financial support for your study. List the grants, grant numbers, and organizations that funded your study, including funding received from your institution. Please note that suppliers of material support, including research materials, should be recognized in the Acknowledgements section rather than in the Financial Disclosure.

ii. State the initials, alongside each funding source, of each author to receive each grant. For example: "This work was supported by the National Institutes of Health (####### to AM; ###### to CJ) and the National Science Foundation (###### to AM)."

iii. State what role the funders took in the study. If the funders had no role in your study, please state: “The funders had no role in study design, data collection and analysis, decision to publish, or preparation of the manuscript.”

iv. If any authors received a salary from any of your funders, please state which authors and which funders

2. For studies involving third-party data, we encourage authors to share any data specific to their analyses that they can legally distribute. PLOS recognizes, however, that authors may be using third-party data they do not have the rights to share. When third-party data cannot be publicly shared, authors must provide all information necessary for interested researchers to apply to gain access to the data. (https://journals.plos.org/plosone/s/data-availability#loc-acceptable-data-access-restrictions)

For any third-party data that the authors cannot legally distribute, they should include the following information in their Data Availability Statement upon submission:

1) A description of the data set and the third-party source

2) If applicable, verification of permission to use the data set

3) Confirmation of whether the authors received any special privileges in accessing the data that other researchers would not have

4) All necessary contact information others would need to apply to gain access to the data

3. Please ensure that your Ethics Statement is available in its entirety at the beginning of your Methods section, under a subheading 'Ethics Statement'.

4. We have noticed that you have uploaded Supporting Information files, but you have not included a list of legends. Please add a full list of legends for your Supporting Information files after the references list.

If the reviewer comments include a recommendation to cite specific previously published works, please review and evaluate these publications to determine whether they are relevant and should be cited. There is no requirement to cite these works unless the editor has indicated otherwise.

**Additional Editor Comments (if provided):**

Both reviewers recognized the value of the study, particularly its contribution to VZV epidemiology in a setting where population-based seroprevalence data remain limited. They also found the study to be broadly technically sound, with appropriate statistical analyses. However, the reviews identify several areas that require substantial clarification and strengthening before the manuscript can meet the journal’s standards for clarity, reproducibility, and interpretation. In particular, the revised manuscript should address the presentation and interpretation of the modeling framework and epidemiological parameters, improve consistency in the reporting of antibody concentrations and figures, clarify data availability, and expand discussion of the limitations and generalizability of the findings.

[Note: HTML markup is below. Please do not edit.]

Reviewers' comments:

Reviewer's Responses to Questions

**Comments to the Author**

1. Does this manuscript meet PLOS Global Public Health’s publication criteria?

Reviewer #1: Yes

Reviewer #2: Yes

**********

2. Has the statistical analysis been performed appropriately and rigorously?

Reviewer #1: Yes

Reviewer #2: Yes

**********

3. Have the authors made all data underlying the findings in their manuscript fully available (please refer to the Data Availability Statement at the start of the manuscript PDF file)?

The PLOS Data policy

Reviewer #1: No

Reviewer #2: Yes

**********

4. Is the manuscript presented in an intelligible fashion and written in standard English?

Reviewer #1: Yes

Reviewer #2: Yes

**********

**Reviewer #1:** This manuscript addresses an important gap in VZV epidemiology in sub-Saharan Africa using robust serological data and appropriate modeling approaches. However, there are minor methodological and reporting limitations, that need clarification. I recommend minor revision before the manuscript can be considered for publication. detailed review comments has been uploaded as attachment.

**Reviewer #2:**  This manuscript examines the seroepidemiology of VZV in Kilifi, Kenya where varicella vaccine is not currently included in the national infant immunization schedule. The study is valuable given the relative scarcity of population-based VZV seroprevalence data from LMICs, despite their importance for understanding disease burden and informing immunization policy decisions. By estimating key epidemiological parameters, the findings may also contribute to understanding transmission dynamics and informing future transmission models, particularly for LMIC settings. Strengths of the study include its population-based seroprevalence estimates derived from large random samples across multiple age groups, as well as the application of catalytic models to estimate key epidemiological parameters from retrospective cross-sectional data. My main concerns relate to the clarity and interpretation of the methodological framework and results presentation; several analytical methods and epidemiological concepts would benefit from additional explanation and context for the readership of PLOS Global Health. The manuscript would also benefit from greater clarity and consistency in the presentation of results and figures, as well as expanded discussion of the study limitations and their implications for generalizability. Overall, this is a valuable study with important contribution, but requires some clarification and improved interpretation/reporting.

Major comments:

1. Clarification of Bayesian estimation framework (lines 167-170): Consider providing additional context for the Bayesian estimation procedures for readers less familiar with these methods. Briefly distinguish MCMC as the general framework from Gibbs sampling as the specific algorithm used and clarify why this approach was applied for parameter estimation. Please also relate the Gelman-Rubin statistic to the overall framework (e.g., “convergence of MCMC chains…”).

2. Interpretation of R0 and its relationship to mean age at infection (lines 171-186): Please define R0 in epidemiological terms and provide additional interpretive context for the relationship R0 = L/A (e.g., younger mean age at infection corresponds to higher transmission intensity and larger R0).

3. HIT and translation to vaccination coverage: The HIT is calculated from R0 estimated based on natural infection. In this context, it’s reasonable that primary vaccine failure is not included. It would be helpful to note in the discussion, though, that this theoretical threshold does not directly translate to vaccination coverage targets, and that vaccine efficacy should be considered when determining the coverage needed to achieve herd immunity.

4. Clarity and consistency of IgG concentrations and figures (Figure 1B, S1A and S1B):

a. Lines 216-222: There appears to be a lack of clarity regarding whether VZV IgG concentrations are reported on the original or log-transformed scale. The text describes “log-transformed VZV IgG antibody” but reports values in IU/mL that appear to correspond to back-transformed geometric mean concentrations, while Figure 1B appears to display log-transformed values (including negative means). Please clarify the scale used for both the statistical summaries and the figure to ensure consistency in interpretation. In addition, the numerical values reported in the text should be checked for consistency with those shown in Figure 1B, as there appear to be minor discrepancies. Also in line 220, “lowest” of what? E.g., Please correct to “lowest concentration of 0.04…”

b. Lines 236-238: Please clarify if these values are averaged across years or referencing a specific year.

c. Lines 242-244: Please describe the pattern (e.g., above the seropositivity threshold).

5. Generalizability to other settings (line 368): The study was conducted in a rural coastal setting, which as mentioned in the limitations section, may limit generalizability. Given that varicella transmission is strongly dependent on contact patterns and age structure, it would be helpful if the authors could discuss how FOI, R), or other estimates might differ in urban or higher density settings.

Minor comments:

• Lines 49-52 are missing citations

• Some in-text citations are missing a space between the text and citation. Please correct. Examples include line 69 and 71.

• Please correct “95%CI” to “95% CI” where it appears throughout the manuscript and in the tables.

• Line 125: a space is needed between “post-stratification” and “(MLRP)”

• Lines 130-131: Please cite the appropriate methodological references (rather than a prior applied study) and provide sufficient detail on the specification of the non-informative priors used (e.g., distributions and variance/hyperparameters) to ensure reproducibility.

• Line 185: Please center-align the equation.

• Table 1: Please add the statistical test conducted (e.g., in footnotes)

• Line 215: Incomplete sentence. Please correct.

• Table 2: For age groups 15 years and over, please either remove or clarify the value that is in parentheses. For example, for age group 15-24, N = 105 (3.3). What is 3.3?

• Figures 3 and 4: Please modify captions to be more descriptive: “…the associated binomial 95% confidence intervals by the black vertical lines”. I assume the gray band around the solid horizontal line (model predictions) is the 95% CI, but please clarify this in the caption.

• Line 268: Please modify to “This corresponds to a decrease in the reproduction number…”

• Line 270: Please specify “The median FOI…”

• Table 3: In the footnote, the terms “first” and “second” catalytic model may be ambiguous. Is this in reference to chronology or to model structure/specifications? Please clarify.

• Lines 293-295: The waning rate is reported as 0.087 per year, but its interpretation is not entirely clear. Please clarify the epidemiological meaning of this parameter: is it the annual rate or hazard of losing immunity, seropositivity, or detectable antibodies?

• Line 328: “Available evidence show” should be “Available evidence shows”

• Please review in-text references to figures and tables and ensure that they are consistent in their typography according to PLOS formatting style. For example, in line 128, Table S2 is unnecessarily bolded. In line 264, the “f” in “(figure 3)…(figure 4)” should be capitalized. And in line 272, “(Table 3)” is unnecessarily bolded and italicized.

**********

what does this mean?). If published, this will include your full peer review and any attached files.

**Do you want your identity to be public for this peer review?**  If you choose “no”, your identity will remain anonymous but your review may still be made public.

For information about this choice, including consent withdrawal, please see our Privacy Policy

Reviewer #1: **Yes:** Josphat Muema

Reviewer #2: **Yes:** Katie Tseng

**********

[NOTE: If reviewer comments were submitted as an attachment file, they will be attached to this email and accessible via the submission site. Please log into your account, locate the manuscript record, and check for the action link "View Attachments". If this link does not appear, there are no attachment files.]

**Figure Resubmissions:**

Attachment Submitted filename: Peer review report_PGPH-D-26-00379.pdf

Attachment Submitted filename: PGPH-D-26-00379-Reviewer1-CommentstoAuthors.docx

# Author comment: Author response to Decision Letter 1

**DOI:** 10.1371/journal.pgph.0006013.r002

24 Jun 2026

Attachment Submitted filename: Response to Reviewers.docx

# Editor report: Decision Letter 1

**DOI:** 10.1371/journal.pgph.0006013.r003

**Authors:** Maria del Pilar Fernandez

25 Jun 2026

PGPH-D-26-00379R1

Trends and Patterns of Varicella Zoster Virus Infection in Kilifi: A Population-based Serosurvey

PLOS Global Public Health

Dear Dr. Sigilai,

Thank you for submitting your manuscript to PLOS Global Public Health. After careful consideration, we feel that it has merit but does not fully meet PLOS Global Public Health’s publication criteria as it currently stands. Therefore, we invite you to submit a revised version of the manuscript that addresses the points raised during the review process.

Please submit your revised manuscript by Jul 25 2026 11:59PM. If you will need more time than this to complete your revisions, please reply to this message or contact the journal office at globalpubhealth@plos.org. When you're ready to submit your revision, log on to https://www.editorialmanager.com/pgph/ and select the 'Submissions Needing Revision' folder to locate your manuscript file.

Please include the following items when submitting your revised manuscript:

- A letter that responds to each point raised by the editor and reviewer(s). You should upload this letter as a separate file labeled 'Response to Reviewers'.
- A marked-up copy of your manuscript that highlights changes made to the original version. You should upload this as a separate file labeled 'Revised Manuscript with Track Changes'.
- An unmarked version of your revised paper without tracked changes. You should upload this as a separate file labeled 'Manuscript'.

Guidelines for resubmitting your figure files are available below the reviewer comments at the end of this letter.

As the corresponding author, your ORCID iD is verified in the submission system and will appear in the published article. PLOS supports the use of ORCID, and we encourage all coauthors to register for an ORCID iD and use it as well. Please encourage your coauthors to verify their ORCID iD within the submission system before final acceptance, as unverified ORCID iDs will not appear in the published article. *Only*  the individual author can complete the verification step; PLOS staff *cannot*  verify ORCID iDs on behalf of authors.

We look forward to receiving your revised manuscript.

Kind regards,

Maria del Pilar Fernandez

Academic Editor

PLOS Global Public Health

**Journal Requirements:**

If the reviewer comments include a recommendation to cite specific previously published works, please review and evaluate these publications to determine whether they are relevant and should be cited. There is no requirement to cite these works unless the editor has indicated otherwise.

Please review your reference list to ensure that it is complete and correct. If you have cited papers that have been retracted, please include the rationale for doing so in the manuscript text, or remove these references and replace them with relevant current references. Any changes to the reference list should be mentioned in the rebuttal letter that accompanies your revised manuscript. If you need to cite a retracted article, indicate the article’s retracted status in the References list and also include a citation and full reference for the retraction notice.

**Additional Editor Comments (if provided):**

Thank you for submitting the revised manuscript and detailed response to the reviewers. The reviewers’ comments have been addressed satisfactorily overall, and the manuscript is now close to being suitable for publication. Before a final decision can be made, please address the following remaining editorial points in a clean revised version.

1. Please revise the conclusion to avoid implying that this study directly measured adult VZV disease burden or demonstrated vaccine impact. The current conclusion states that the data show “a significant VZV burden in adults which can be prevented by a childhood vaccine programme.” This wording should be tempered because the study measured VZV IgG seroprevalence, susceptibility, and modelled force of infection, but did not directly measure clinical disease burden, complications, hospitalizations, mortality, congenital varicella syndrome, or vaccine effectiveness.

Please revise this language so that the conclusion is proportionate to the data presented. For example, instead of:

“Taken together, seroepidemiological and modelling data show a significant VZV burden in adults which can be prevented by a childhood vaccine programme.”

Please consider revising to:

“Taken together, the seroepidemiological and modelling data identify a substantial susceptibility gap extending into adolescence and adulthood, suggesting the potential for continued VZV infection at older ages. These findings support the need for additional studies of VZV morbidity burden, vaccine impact, cost-effectiveness, and programmatic feasibility to inform varicella vaccine policy in Kenya.”

2. Please ensure that the final submitted manuscript is fully clean, with all tracked-change artifacts, duplicated text, editorial comments, formatting remnants, and inconsistent figure/table references removed. For example, the sentence explaining the relationship between R₀ and mean age at infection currently appears to contain a corrupted phrase: “populations experiencing higher transvalues. Uncertaintytend…” Please revise this sentence so that it reads clearly and grammatically.

3. Please revise the figures to improve readability and ensure they meet publication-quality standards.

For Figure 1, please ensure that all survey years are fully visible on the x-axis and that the axis labels, panel labels, and seropositivity threshold line are legible at final publication size. Please also confirm that the caption clearly distinguishes between the GMC estimates reported in the text on the original IU/mL scale and the log-transformed IgG concentrations shown in panel B.

For Figure 2, please improve the spacing of the faceted panels and x-axis age-category labels. In the current version, the age-category labels are crowded and the rightmost panel appears difficult to read. Please ensure that each survey year panel is fully visible, that the age categories are not clipped or overlapping, and that the seropositivity threshold line is clearly labeled or explained in the caption.

For Figure 3, please ensure that all panel labels, age-axis labels, observed points, confidence intervals, model-predicted line, and credible interval shading are legible. Please also confirm that the figure caption clearly states what the black points, black vertical lines, solid line, and grey shaded area represent.

For Figure 4, please consider simplifying the presentation of the adult seroprevalence model because the current figure is visually crowded, with many points and wide confidence intervals. One option would be to present age-banded estimates rather than many individual-age estimates. Another option would be to move the current detailed figure to the supplement and include a clearer main-text figure showing seroprevalence by adult age band with model estimates and uncertainty. At minimum, please improve the scaling and spacing so the data points, uncertainty intervals, model line, and grey credible interval band can be interpreted clearly.

4. Please review the data availability statement and ensure that it clearly explains the process for accessing the underlying data. If the data cannot be deposited publicly because of ethical, legal, or governance restrictions, please state this explicitly and provide sufficient detail on how qualified researchers may request access.

[Note: HTML markup is below. Please do not edit.]

**Reviewers' comments:**

[NOTE: If reviewer comments were submitted as an attachment file, they will be attached to this email and accessible via the submission site. Please log into your account, locate the manuscript record, and check for the action link "View Attachments". If this link does not appear, there are no attachment files.]

**Figure Resubmissions:**

# Author comment: Author response to Decision Letter 2

**DOI:** 10.1371/journal.pgph.0006013.r004

9 Jul 2026

Attachment Submitted filename: Response to Reviewers_1.docx

# Editor report: Decision Letter 2

**DOI:** 10.1371/journal.pgph.0006013.r005

**Authors:** Maria del Pilar Fernandez

19 Jul 2026

Trends and Patterns of Varicella Zoster Virus Infection in Kilifi: A Population-based Serosurvey

PGPH-D-26-00379R2

Dear Mr. Sigilai,

We are pleased to inform you that your manuscript 'Trends and Patterns of Varicella Zoster Virus Infection in Kilifi: A Population-based Serosurvey' has been provisionally accepted for publication in PLOS Global Public Health.

Before your manuscript can be formally accepted you will need to complete some formatting changes, which you will receive in a follow up email. A member of our team will be in touch with a set of requests.

Please note that your manuscript will not be scheduled for publication until you have made the required changes, so a swift response is appreciated.

IMPORTANT: The editorial review process is now complete. PLOS will only permit corrections to spelling, formatting or significant scientific errors from this point onwards. Requests for major changes, or any which affect the scientific understanding of your work, will cause delays to the publication date of your manuscript.

If your institution or institutions have a press office, please notify them about your upcoming paper to help maximize its impact. If they'll be preparing press materials, please inform our press team as soon as possible -- no later than 48 hours after receiving the formal acceptance. Your manuscript will remain under strict press embargo until 2 pm Eastern Time on the date of publication. For more information, please contact globalpubhealth@plos.org.

Thank you again for supporting Open Access publishing; we are looking forward to publishing your work in PLOS Global Public Health.

Best regards,

Maria del Pilar Fernandez

Academic Editor

PLOS Global Public Health
